# Genome‐wide association analysis of natural variation in seed tocochromanols of barley

**DOI:** 10.1002/tpg2.20039

**Published:** 2020-09-28

**Authors:** Ramamurthy Mahalingam, Ahmad H. Sallam, Brian J. Steffenson, Jason D. Fiedler, Jason G. Walling

**Affiliations:** ^1^ USDA‐ARS Cereal Crops Research Unit 502 Walnut Street Madison WI 53726 USA; ^2^ Department of Plant Pathology Univ. of Minnesota St. Paul MN 55108 USA; ^3^ USDA‐ARS Cereal Crops Research Unit 1616 Albrecht Blvd Fargo ND 58102 USA

## Abstract

Tocochromanols (tocols for short), commonly called Vitamin E, are lipid‐soluble plant antioxidants vital for regulating lipid peroxidation in chloroplasts and seeds. Barley (*Hordeum vulgare* L.) seeds contain all eight different isoforms of tocols; however, the extent of natural variation in their composition and their underlying genetic basis is not known. Tocol levels in barley seeds were quantified in diverse *H. vulgare* panels comprising 297 wild lines from a diversity panel and 160 cultivated spring‐type accessions from the mini‐core panel representing the genetic diversity of the USDA barley germplasm collection. Significant differences were observed in the concentration of tocols between the two panels. To identify the genes associated with tocols, genome‐wide association analysis was conducted with single nucleotide polymorphisms (SNPs) from Illumina arrays for the mini‐core panel and genotyping‐by‐sequencing for the wild barley panel. Forty unique SNPs in the wild barley and 27 SNPs in the mini‐core panel were significantly associated with various tocols. Marker–trait associations (MTAs) were identified on chromosomes 1, 6, and 7 for key genes in the tocol biosynthesis pathway, which have also been reported in other studies. Several novel MTAs were identified on chromosomes 2, 3, 4 and 5 and were found to be in proximity to genes involved in the generation of precursor metabolites required for tocol biosynthesis. This study provides a valuable resource for barley breeding programs targeting specific isoforms of seed tocols and for investigating the physiological roles of these metabolites in seed longevity, dormancy, and germination.

**Abbreviations**
AT: α‐tocopherol
AT3: α‐tocotrienol
BT: β‐tocopherol
BT3: β‐tocotrienol
CRAL‐TRIO: Cellular retinaldehyde‐binding protein (CRAL) and triple functional domain protein (TRIO) guanine exchange factor
DT: δ‐tocopherol
DT3: δ‐tocotrienol
DXP: 1‐Deoxy‐*d*‐xylulose‐5‐phosphate synthase
GGDP: Geranylgeranyl diphosphate
GGDR: Geranylgeranyl diphosphate reductase
GT: γ‐tocopherol
GT3: γ‐tocotrienol
GWAS: Genome‐wide association study
HGGT: Homogentysyl geranylgeranyl transferase
LD: Linkage disequilibrium
MEP: Methylerythritol phosphate
MTA: Marker–trait association
PCA: Principal component analysis
QTL: Quantitative trait locus
QVE: Quantitative trait locus associated with Vitamin E
SNP: Single nucleotide polymorphism
TAT: Tyrosine amino transferase
TBP: tocopherol binding protein
TMT: Tocopherol methyltransferase
Tocol: Tocochromanol
*VTE*
: Vitamin E gene
WBDC: Wild Barley Diversity Collection.

## 1 INTRODUCTION

**FIGURE 1:**
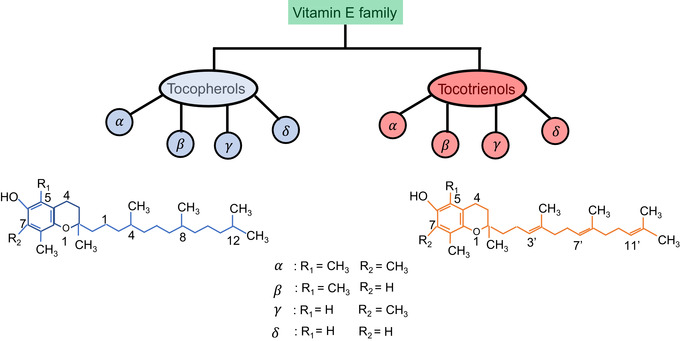
Structure of tocochromanols

Vitamin E includes a family of eight compounds that are biochemically referred to as tocochromanols (tocols for in short) (Munne‐Bosch & Alegre, 2002). These tocols consist of a chromanol ring and a polyprenyl side chain. The chromanol ring is derived via the shikimate pathway from homogentisate. Depending on the origin of the prenyl side chain, tocols are divided into two subgroups: tocopherols and tocotrienols. In tocopherols, the 15‐C tail attached to the chromanol ring is derived from phytyl diphosphate; in tocotrienols, it originates from geranylgeranyl diphosphate (GGDP). Further, depending on the number and position of the methyl groups on the chromanol ring, there are four different isoforms of tocopherol and tocotrienols. The α isoforms have three methyl groups at positions 5, 7, and 8 in the chromanol ring, whereas the β and γ isoforms have a methyl group at the eighth position and a methyl group at the fifth position or seventh position respectively. The δ isoforms have only one methyl group at the eighth position on their chromanol ring (Figure 1).

Vitamin E compounds are lipid‐soluble and synthesized in the plastids of plants, algae, and cyanobacteria (Schultz, Soll, Fiedler, & Schulzesiebert, 1985). Alpha‐tocopherol (AT), a major tocol isoform in green plant tissues, is involved in preserving the integrity of photosynthetic membranes by preventing lipid peroxidation (Munne‐Bosch, 2005). Tocopherols play an important role in seed longevity by limiting the oxidation of storage lipids in the seeds and protecting seeds from reactive oxygen species (Sattler, Gilliland, Magallanes‐Lundback, Pollard, & DellaPenna, 2004). However, γ‐tocopherol (GT) may negatively impact seed germination and seedling development through modulating nitric oxide signaling (Desel & Krupinska, 2005). In barley, seed longevity and germination are important factors that directly have a bearing not only on the growers but also their major stakeholders, the malting and brewing industries.

The natural abundance and high Vitamin E activity of tocopherols, especially AT, (Bieri & Evarts, 1974), combined with the discovery of the AT transfer protein in humans (Arita et al., 1995; Hosomi et al., 1997), have led to a biased focus on this particular isoform. This is even more evident in how the recommended dietary allowance for Vitamin E is issued only for AT, since it is the only form maintained in the plasma. Tocotrienols have recently received considerable attention for their various biological properties such as their antioxidative (Serbinova, Kagan, Han, & Packer, 1991), antihypercholesterolemic (Qureshi, Sami, Salser, & Khan, 2002), anticancer (Goh, Hew, Norhanom, & Yadav, 1994; Takahashi & Loo, 2004), and neuroprotective activity (Khanna et al., 2003). These findings suggest that tocotrienols have a wide range of physiological functions; therefore, developing plants that could synthesize high concentrations of these compounds would be useful for nutraceutical applications. Tocotrienols are found almost exclusively in the seeds of cereal grains and palm seeds (Khan, Ahsan, Siddiqui, & Siddiqui, 2015). All four isoforms of tocotrienol have been reported in developing barley grains (Falk, Krahnstover, van der Kooij, Schlensog, & Krupinska, 2004) and hence provide an excellent model for further analysis of these metabolites.

**Core Ideas**

- Extensive natural variation for seed tocochromanol levels exists in barley
- Alpha‐tocopherol levels differed significantly between wild and domesticated barley
- Diverse panels & high‐density genotyping enabled marker–trait associations (MTAs)
- MTAs were identified for all the primary tocochromanol biosynthesis pathway genes

The cloning of Vitamin E biosynthesis genes (*VTE* genes) in the model plant *Arabidopsis thaliana* (L.) Heynh. allowed the core tocol pathway to be engineered for improved nutritional content and composition in various plants (Collakova & DellaPenna, 2003; Hunter & Cahoon, 2007; Karunanandaa et al., 2005; Kumar et al., 2005; Lu, Rijzaani, Karcher, Ruf, & Bock, 2013; Savidge et al., 2002; Shintani & DellaPenna, 1998; Zhang et al., 2013). Efforts to identify the genetic loci associated with tocopherol biosynthesis have been reported for rice (*Oryza sativa* L.) (Sookwong et al., 2009), oats (*Avena sativa* L.) (Jackson et al., 2008), maize (Chander et al., 2008; Baseggio et al., 2019), and barley (Graebner et al., 2015; Oliver, Islamovic, Obert, Wise, & Herrin, 2014). In most of these studies, the major quantitative trait loci (QTLs) identified were found to be associated with one or two key genes in the tocol biosynthetic pathway.

In this study, we have analyzed the seed tocol concentrations from two diverse panels of *H. vulgare*: the Wild Barley Diversity Collection (WBDC) panel consisting of 297 *H. vulgare* ssp. *spontaneum* accessions (Sallam et al., 2017) and the mini‐core panel consisting of 160 cultivated spring‐type *H. vulgare* ssp. *vulgare* accessions that represents the genetic diversity of the USDA barley icore collection (Munoz‐Amatriain et al., 2013). A genome‐wide association study (GWAS) was undertaken to identify the genetic loci associated with each of the eight different isoforms of Vitamin E, tocopherols (the total of the four tocopherol isoforms), tocotrienols (total of the four tocotrienol isoforms) and total tocols (the total of all eight isoforms). The vast phenotypic variability in the concentration of these metabolites in the two highly diverse germplasm panels, together with the use of high‐density SNP marker mapping, enabled the identification of significant MTAs for genes involved in tocol biosynthesis. Several novel QTLs that accounted for a significant amount of the phenotypic variation of tocols were identified in close proximity to genes in the biosynthesis pathway of tocol precursor metabolites.

## 2 MATERIALS AND METHODS

### 2.1 Plant materials

The mini‐core panel of cultivated barley comprises 184 accessions as previously described (Munoz‐Amatriain et al., 2013). Of the 184 accessions in the mini‐core panel, 160 spring types were selected. The seeds were obtained from the USDA‐ARS National Small Grains Collection (Aberdeen, ID) and then grown in a greenhouse at the USDA‐ARS Cereal Crops Research Unit in 2016 and 2017. Plants were grown in a potting mix consisting of sand, peat moss, and vermiculite (2:1:1). Osmocote 14–14–14 N–P–K (Scott's Co, Marysville, OH), was added to the potting mix (2 g per pot). Plants were watered regularly and maintained at 18 to 22 °C with 16 h of light (380 μmol m^−2^ s^−1^) until harvest. Spikes were harvested at maturity and the seeds were collected after threshing (LT15 laboratory thresher, Haldrup, Poneto, IN). The 297 WBDC accessions used in this study were collected from two field plantings (2005–2006 and 2015–2016) at the University of California, Davis. Seeds from these two field collections were stored in a cold room at the University of Minnesota and have previously been used in a GWAS of stem rust resistance (Sallam et al., 2017) and seed β‐glucan content.

### 2.2 Tocochromanol assay and phenotypic data analysis

Barley seeds were ground in a Retsch ZM‐1 mill (Retsch, Haan, Germany) and extracted via a modified hot saponification and extraction method (Fratianni, Caboni, Irano, & Panfili, 2002) (Supplementary File S1). Identification of individual forms was based on retention time, and quantification was based on standard curves developed from authentic tocopherols (Metraya LLC, Pleasant Gap, PA) (Supplemental Figure S1). Tocotrienols, which have essentially the same fluorescent properties as their corresponding tocopherols (Thompson & Hatina, 1979), were quantified with the same standard curves.

The eight tocols identified in this study were AT, β‐tocopherol (BT), GT, δ‐tocopherol (DT), α‐tocotrienol (AT3), β‐tocotrienol (BT3), γ‐tocotrienol (GT3), and δ‐tocotrienol (DT3). Additionally, the following three measurements were calculated with the data from the individual isoforms: total tocotrienols, total tocopherols, and total tocols. Within‐line variance and between‐line variances for each of the tocol traits from the two replications were used to estimate broad‐sense heritability in the two populations.

### 2.3 Single nucleotide polymorphism genotyping

Approximately 200 mg of leaf tissue from each accession of the mini‐core panel was used for DNA isolation via standard extraction methods in the presence of RNAse A. The integrity and concentration of DNA were analyzed with a Nanodrop 2000 (ThermoFisher, Waltham, MA). The 50k Illumina Infinium iSelect SNP genotyping array (Bayer et al., 2017) was used for genotyping the 160 barley mini‐core accessions at the North Central Small Grains Genotyping facility (USDA‐ARS Edward T. Schafer Agricultural Research Center, Fargo, ND). Single nucleotide polymorphism alleles were called by GenomeStudio Genotyping Module Version 2.04 (Illumina, San Diego, CA) with the default parameters to the de novo clustering algorithm. Markers with poorly resolved genotype clusters (<15%) were altered so that the cluster locations represented the proper genotype. Artifacts of heterozygous genotypes were manually converted to “missing” if A and B genotypes were present. For the WBDC panel, genotyping‐by‐sequencing marker data were generated as described previously (Sallam et al., 2017). For both the mini‐core collection and the WBDC panel, physical marker positions were used (Mascher et al., 2017).

### 2.4 Mini‐core panel structure analysis

Pairwise genetic distances among all accessions was calculated for all markers as 1 – identity‐by‐state. With the use of the distance matrix, K‐means clustering was performed on the 160 accessions of the barley mini‐core panel with 5,000 iterations and five subpopulations in line with the previous study (Munoz‐Amatriain et al., 2013). To investigate the effect of population structure on GWAS, principal component analysis (PCA) and pairwise genetic similarity was performed in R (http://www.R‐project.org/, accessed 7 Aug. 2020) on the 50k SNP marker data. Subpopulations identified via K‐means clustering were visualized on the PCA plot.

### 2.5 Genome‐wide association study

To identify markers associated with the eight individual tocol isoforms, tocopherols, tocotrienols, and total tocols, GWAS was conducted by applying a mixed linear model that accounted for population structure and kinship in the rrBLUP package (Endelman, 2011). To account for structure, the first two PCAs were used as a covariate in the GWAS model. To account for genetic kinship, genetic relatedness was fitted as a random covariate in the mixed linear model. In order to reduce redundancy in declaring MTAs, several filters were included in this analysis. A highly stringent cutoff that used the lowest 0.0001 percentile of the distribution of *p‐*values for each trait was tested. Secondly, haplotype blocks were generated from the adjacent marker's linkage disequilibrium (LD) (estimated as *r^2^*) in the significant segments, with an LD level of ≥0.2. Only the most significant SNP within a haplotype block was selected. Thirdly, to account for the large number of multiple tests involved in this analysis, a high false discovery rate threshold of 0.0005 was used as a cutoff for identifying significant markers. A Manhattan plot was generated for each tocol isoform and the threshold for detecting significant SNP markers was represented by a horizontal dashed red line. The phenotyic variance (*R^2^*) explained by each MTA was calculated as described earlier (Sallam et al., 2017).

A sliding window of 50 adjacent markers was used to characterize the LD in the mini‐core population. These sliding window LD estimates of *r^2^* were plotted against the physical position. A locally weighted scatter plot smoother was fitted in JMP Version 11.2 (SAS Institute Inc., Cary, NC) to visualize the LD changes with the physical positions.

### 2.6 Gene annotations

Information about genes associated with the tocol biosynthetic pathway and their Ensembl gene identifiers were obtained from a recently published study (Schuy et al., 2019). The physical positions of the tocol biosynthesis pathway genes and the significantly associated SNP markers in proximity to these genes were determined at bp resolution and displayed on a physical barley chromosome map. On the basis of the LD decay patterns, proximity was defined as within 45 Mb for the mini‐core population (Supplemental Figure S2) and within 13 Mb for the WBDC (Sallam et al., 2017). The significant marker loci identified by GWAS were called quantitative Vitamin E loci (QVEs) in line with the description for genetic loci associated with Vitamin E in *A. thaliana* (Gilliland et al., 2006).

In order to identify other genes associated with the tocol pathway, the Ensembl database was used to retrieve all stable gene identifiers within a 45‐Mb window surrounding each new QVE identified in the mini‐core population and a 12‐Mb window for the novel QVEs identified in the WBDC panel. Their corresponding protein sequences and protein domain information were retrieved from the Ensembl database (https://plants.ensembl.org/Hordeum_vulgare/Info/Index, accessed 5 Aug. 2020). On the basis of the protein domains, we manually curated several of the genes in these regions using information from the Interpro (https://www.ebi.ac.uk/interpro/, accessed 5 Aug. 2020), Uniprot (https://www.uniprot.org/, accessed 5 Aug. 2020), Conserved Domain Database (https://www.ncbi.nlm.nih.gov/cdd/, accessed 5 Aug. 2020), and Prosite (https://prosite.expasy.org/, accessed 5 Aug. 2020) databases. Protein sequence alignments were conducted by the TCOFFEE program (http://tcoffee.crg.cat/apps/tcoffee/do:expresso, accessed 5 Aug. 2020). The gene expression profiles of some of the interesting genes associated with significant MTAs were retrieved from the Barlex database (https://apex.ipk-gatersleben.de/apex/f?p=284:10::::::, accessed 7 Aug. 2020)

## 3 RESULTS

### 3.1 Phenotypic data

**FIGURE 2:**
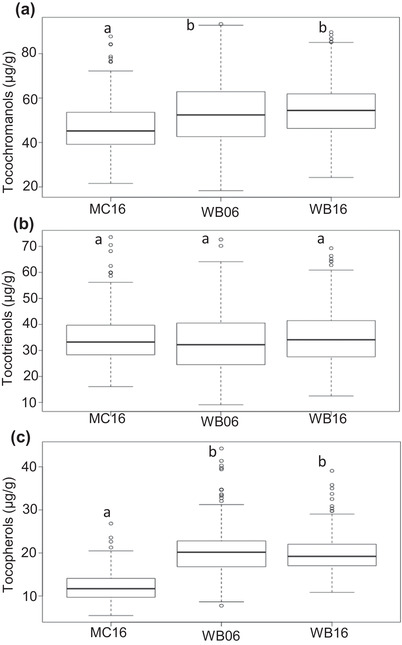
Whisker plots of (a) total tocochromanols, (b) tocotrienols and (c) tocopherols in the mini‐core panel (MC16) and the wild barley panel over two different growing seasons (WB06 and WB16). The box represents the upper and lower quartiles of the distribution and the thick line inside the box represents the median value. The dotted lines extending from the box (whiskers) represent the variability outside the upper and lower quartiles. The circles represent the accessions whose values are outside the range of the normal distribution

The extent of phenotypic variation in the total tocol levels between the wild barley and the mini‐core panels was analyzed. Broad‐sense heritability estimates based on a line‐mean basis for each of the tocol isoforms ranged from 0.93 to 0.98 for the mini‐core population. In the WBDC panel, the average heritability ranged between 0.82 to 0.97 (Table 1). The two biological replications of the seed samples from the wild barley panel indicated that the median values between these replicates were very close (∼52–54 μg g^–1^) (Figure 2a, Supplemental Table S1).

**TABLE 1:** Heritability of tocochromanol traits in the mini‐core and Wild Barley Diversity Collection (WBDC) populations

	Population mean		Within‐line variance		Among‐lines variance		Broad‐sense heritability	
Trait	Mini‐core	WBDC	Mini‐core	WBDC	Mini‐core	WBDC	Mini‐core	WBDC
AT^a^	6.4	13.4	0.080	0.63	1.1	6.8	0.93	0.87
BT	0.6	1.2	0.004	0.02	0.1	0.14	0.96	0.82
GT	4.7	4.9	0.067	0.09	2.5	2.98	0.97	0.97
DT	0.3	0.5	0.001	0.002	0.1	0.045	0.98	0.96
AT3	19.3	23.3	0.508	0.83	16.3	30.2	0.96	0.97
BT3	4.1	2.2	0.024	0.02	2.1	0.57	0.98	0.97
GT3	10.3	8.1	0.126	0.09	7.1	4.2	0.98	0.97
DT3	0.9	0.4	0.001	0.002	0.1	0.05	0.99	0.88
TTC	46.7	54.0	2.419	2.76	55.7	127.7	0.96	0.97

^a^ AT, α‐tocopherol; AT3, α‐tocotrienol; BT, β‐tocopherol; BT3, β‐tocotrienol; GT, γ‐tocopherol; GT3, γ‐tocotrienol; DT, δ‐tocopherol; DT3, δ‐tocotrienol; TTC, total tocochromanols

There were a few accessions from the 2016 season that showed levels above the upper quartile of the plot. Interestingly, in the mini‐core panel, the median value of total tocols was lower (∼44 μg g^–1^) than the WBDC panel (Supplemental Table S2). The upper quartile of the distribution was also significantly lower (70 μg g^–1^) than the WBDC's distribution (85–90 μg g^–1^) (Figure 2a). The lower quartile of the distribution was similar between the two panels.

Next, we examined if the variation in the tocols in the two panels was attributable to tocotrienols or tocopherols. The median values of the distribution of tocotrienols between the panels as well as the upper and lower quartiles were not significantly different (∼32–34 μg g^–1^) (Figure 2b). However, in the mini‐core panel, the number of accessions that were above the upper extreme (represented by the whiskers in Figure 2) was higher than in the WBDC panel. On the contrary, the tocopherol levels in the WBDC panel were nearly twice the amount observed in the mini‐core panel (Figure 2c). A significant number of accessions in the WBDC panel with tocopherol levels exceeded the upper extremes compared with the mini‐core panel.

**FIGURE 3:**
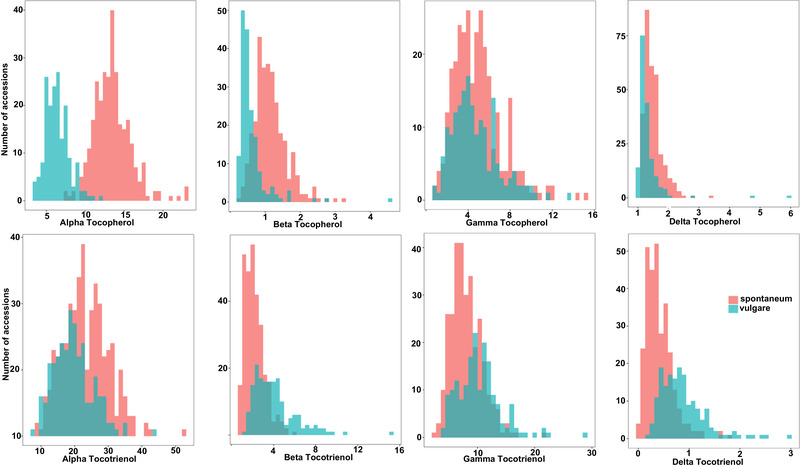
Distribution of the four different tocopherols (α, β, γ, and δ) and four different tocotrienols in the WBDC and mini‐core panels. The values for the wild barley panel are the pooled means over two seasons. The overlap in the distribution between the two panels is shown as a darker green color

The distribution of each isoform of tocopherol and tocotrienol was examined (Figure 3). The abundance of the tocopherol and tocotrienol isoforms in the barley seeds was in this order: α > γ > β > δ. The most abundant isoform was AT3 in both panels. The most significant difference between the panels was observed in AT, which had two distinct distribution patterns: the mini‐core panel was skewed towards lower concentrations, whereas the WBDC panel exhibited higher concentrations of this isoform. This skewness in the distribution between the WBDC and mini‐core panels was much less pronounced for other tocopherols. In contrast to AT, the distribution of AT3 between the WBDC and mini‐core panels showed a significant overlap. Compared with the WBDC panel, the mini‐core panel showed a slight right skew in the distribution of BT3 and DT3. Similar to the distribution of GT, the distribution of GT3 showed a significant overlap between the two panels.

**FIGURE 4:**
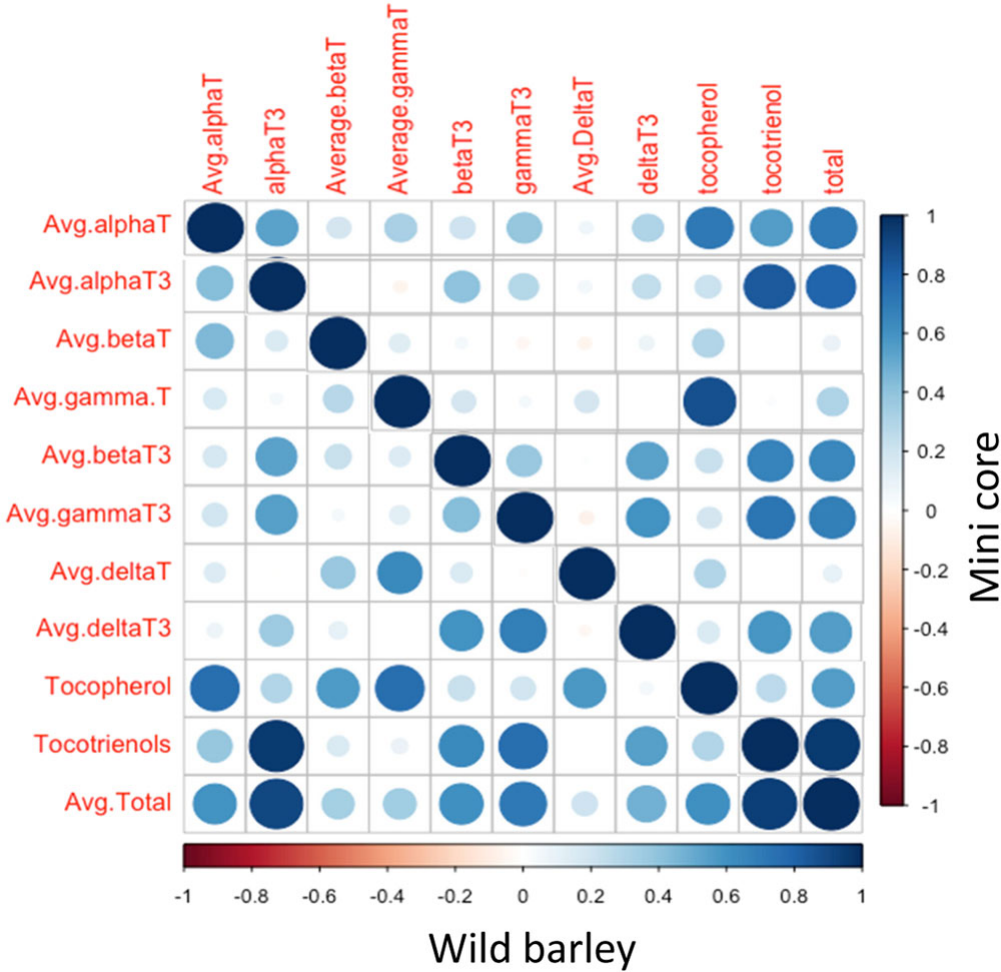
Correlation analysis of the eight tocochromanol isoforms, tocopherols, and tocotrienols in the Wild Barley Diversity Collection (WBDC) and mini‐core panel

In order to get a better appreciation for the contribution of each of the tocol isoforms to total tocol levels, a correlation analysis was undertaken for the two panels separately (Figure 4). In this analysis, it was clear that vast majority of the correlations were positive and only a few minor negative correlations were noted among BT, GT3, and DT in the mini‐core panel and between DT3 and DT in the WBDC panel. The contributions of the isoforms to the total tocols in the two panels showed some subtle differences. In the WBDC panel, the relative levels of the tocol isoforms was AT3 > GT3 > AT = BT3 > DT3 > BT = GT > DT; in the mini‐core panel, the hierarchy of isoform distributions was AT3 > AT ≥ GT3 = BT3 > DT3 > GT > DT > BT (Table 2).

**TABLE 2:** Accessions from the Wild Barley Diversity Collection (WBDC) and mini‐core panels with highest or lowest amounts of tocochromanol isoforms

Accession^a^	AT^b^	AT3	BT	GT	BT3	GT3	DT	DT3	TT	TT3	TTC
WBDC 309	**23.25** ^c^	29.96	1.66	3.58	1.39	7.41	0.51	0.18	29.00	38.94	67.93
WBDC 036	7.44	11.77	1.00	4.16	1.54	3.48	0.31	0.10	12.91	16.89	29.79
MC011	**11.86**	24.97	1.06	11.43	3.56	9.00	0.86	0.80	25.21	38.33	63.54
MC023	3.35	10.29	0.30	2.06	1.31	5.50	0.08	0.32	5.78	17.43	23.21
WBDC 276	16.80	**52.00**	1.51	2.17	1.61	17.20	0.27	0.61	20.75	71.43	92.17
WBDC 067	11.73	8.84	1.42	1.88	2.08	2.35	2.33	0.19	17.37	13.46	30.83
MC004	6.38	**35.32**	1.13	3.94	6.16	11.98	0.14	0.82	11.59	54.28	65.86
MC005	4.16	7.90	0.44	9.16	4.00	6.32	0.19	0.56	13.95	18.79	32.74
WBDC 197	14.62	14.25	**3.22**	2.38	1.06	4.25	0.28	0.26	20.51	19.81	40.32
WBDC 156	9.39	30.03	0.29	2.83	3.09	8.89	0.13	0.33	12.65	42.34	54.99
MC158	5.90	14.52	**4.61**	4.55	2.10	6.54	0.18	0.46	15.24	23.61	38.85
MC0151	5.76	21.14	0.26	4.53	2.69	6.21	0.15	0.43	10.69	30.46	41.16
WBDC 011	10.51	24.96	1.76	**15.17**	1.17	6.43	0.82	0.27	28.27	32.83	61.10
WBDC 293	11.34	27.23	1.00	0.94	2.38	13.39	0.22	1.15	13.51	44.15	57.66
MC183	5.83	18.99	0.26	**13.63**	15.28	10.09	0.98	0.34	20.70	44.69	65.40
MC 146	5.44	19.46	0.42	0.72	4.32	9.04	0.02	0.72	6.61	33.55	40.16
WBDC 344	17.28	37.70	1.33	5.94	**6.08**	10.17	0.34	0.94	24.89	54.89	79.77
WBDC 317	12.78	12.73	1.20	8.05	0.66	5.57	0.70	0.03	22.73	18.99	41.72
MC183	5.83	18.99	0.26	13.63	**15.28**	10.09	0.98	0.34	20.70	44.69	65.40
MC023	3.35	10.29	0.30	2.06	1.31	5.50	0.08	0.32	5.78	17.43	23.21
WBDC 075	10.41	17.28	0.44	11.45	2.06	**21.27**	0.40	1.83	22.70	42.44	65.14
WBDC 067	11.73	8.84	1.42	1.88	2.08	2.35	2.33	0.19	17.37	13.46	30.83
MC037	10.60	32.04	1.30	2.03	8.38	**28.70**	0.06	2.94	13.99	72.05	86.04
MC002	6.25	18.05	0.59	2.39	3.64	3.99	0.11	0.38	9.35	26.05	35.40
WBDC 067	11.73	8.84	1.42	1.88	2.08	2.35	**2.33**	0.19	17.37	13.46	30.83
WBDC 139	9.39	14.28	0.49	2.20	0.95	5.48	0.08	0.16	12.16	20.87	33.03
MC009	4.28	17.22	0.28	1.49	2.56	8.55	**4.98**	0.88	11.03	29.21	40.24
MC154	3.57	13.48	0.26	2.98	2.18	4.90	0.01	0.41	6.82	20.96	27.78
WBDC 075	10.41	17.28	0.44	11.45	2.06	21.27	0.40	**1.83**	22.70	42.44	65.14
WBDC 154	12.03	13.51	0.78	6.98	0.85	4.49	0.61	0.03	20.39	18.87	39.26
MC037	10.60	32.04	1.30	2.03	8.38	28.70	0.06	**2.94**	13.99	72.05	86.04
MC112	4.85	15.18	0.30	1.18	2.42	9.35	0.60	0.19	6.93	27.14	34.07
WBDC 312	22.11	24.68	2.38	14.23	2.25	7.55	1.14	0.47	**39.87**	34.95	74.82
WBDC 159	7.75	9.15	0.51	1.29	0.85	3.66	0.08	0.09	9.64	13.75	23.39
MC011	11.86	24.97	1.06	11.43	3.56	9.00	0.86	0.80	**25.21**	38.33	63.54
MC039	3.59	12.89	0.47	1.57	4.87	8.08	0.06	0.94	5.69	26.78	32.47
WBDC 276	16.80	52.00	1.51	2.17	1.61	17.20	0.27	0.61	20.75	**71.43**	92.17
WBDC 067	11.73	8.84	1.42	1.88	2.08	2.35	2.33	0.19	17.37	13.46	30.83
MC037	10.60	32.04	1.30	2.03	8.38	28.70	0.06	2.94	13.99	**72.05**	86.04
MC023	3.35	10.29	0.30	2.06	1.31	5.50	0.08	0.32	5.78	17.43	23.21
WBDC 276	16.80	52.00	1.51	2.17	1.61	17.20	0.27	0.61	20.75	71.43	**92.17**
WBDC 159	7.75	9.15	0.51	1.29	0.85	3.66	0.08	0.09	9.64	13.75	23.39
MC037	10.60	32.04	1.30	2.03	8.38	28.70	0.06	2.94	13.99	72.05	**86.04**
MC023	3.35	10.29	0.30	2.06	1.31	5.50	0.08	0.32	5.78	17.43	23.21
WB avg.	13.43	23.29	1.15	4.91	2.18	8.10	0.50	0.41	20.00	33.98	53.98
MC avg.	6.42	19.33	0.61	4.73	4.08	10.27	0.34	0.89	12.10	34.57	46.67

^a^ Lines from the Wild Barley Diversity Collection are designated as WB and lines from the mini‐core collection are designated as MC

^b^ AT, α‐tocopherol; AT3, α‐tocotrienol; BT, β‐tocopherol; BT3, β‐tocotrienol; GT, γ‐tocopherol; GT3, γ‐tocotrienol; DT, δ‐tocopherol; DT3, δ‐tocotrienol; TTC, total tocochromanols; TT, total tocopherols; TT3, total tocotrienols.

^c^ Bold font represents the highest concentration of the particular tocol isoform measured in the barley lines; underlining indicates the lowest concentration.

### 3.2 Genotypic analyses and LD of the mini‐core panel

In the Infinium 50k arrays, 44,041 SNPs were screened after removing the genotype clusters with poor scores. In this set, 232 SNPs had no calls and were removed from further analysis. After screening for a minor allele frequency of 5%, 32,942 markers were used for the final analyses.

The LD was estimated from these SNP markers, the number of SNPs for each chromosome, and the average adjacent marker LD for each chromosome. The average LD for the chromosomes ranged from 0.42 (2H) to 0.51 (1H) with an overall average LD of 0.46 (Table 3). With a sliding window of 50 markers, LD as *r* ^2^ decayed to 0.22 at ∼45 Mb and LD never reduced below 0.1 (Supplemental Figure S2).

**TABLE 3:** Distribution of linkage disequilibrium (LD) in the genome of *H. vulgare* accessions in the mini‐core population

	Sites	
Chromosome	*n*	Average LD
1H	3,690	0.51
2H	5,418	0.42
3H	5,048	0.49
4H	3,906	0.48
5H	6,108	0.43
6H	4,045	0.43
7H	4,727	0.48
Genome	32,942	0.46

### 3.3 Population structure and genetic relatedness in the mini‐core panel

**FIGURE 5:**
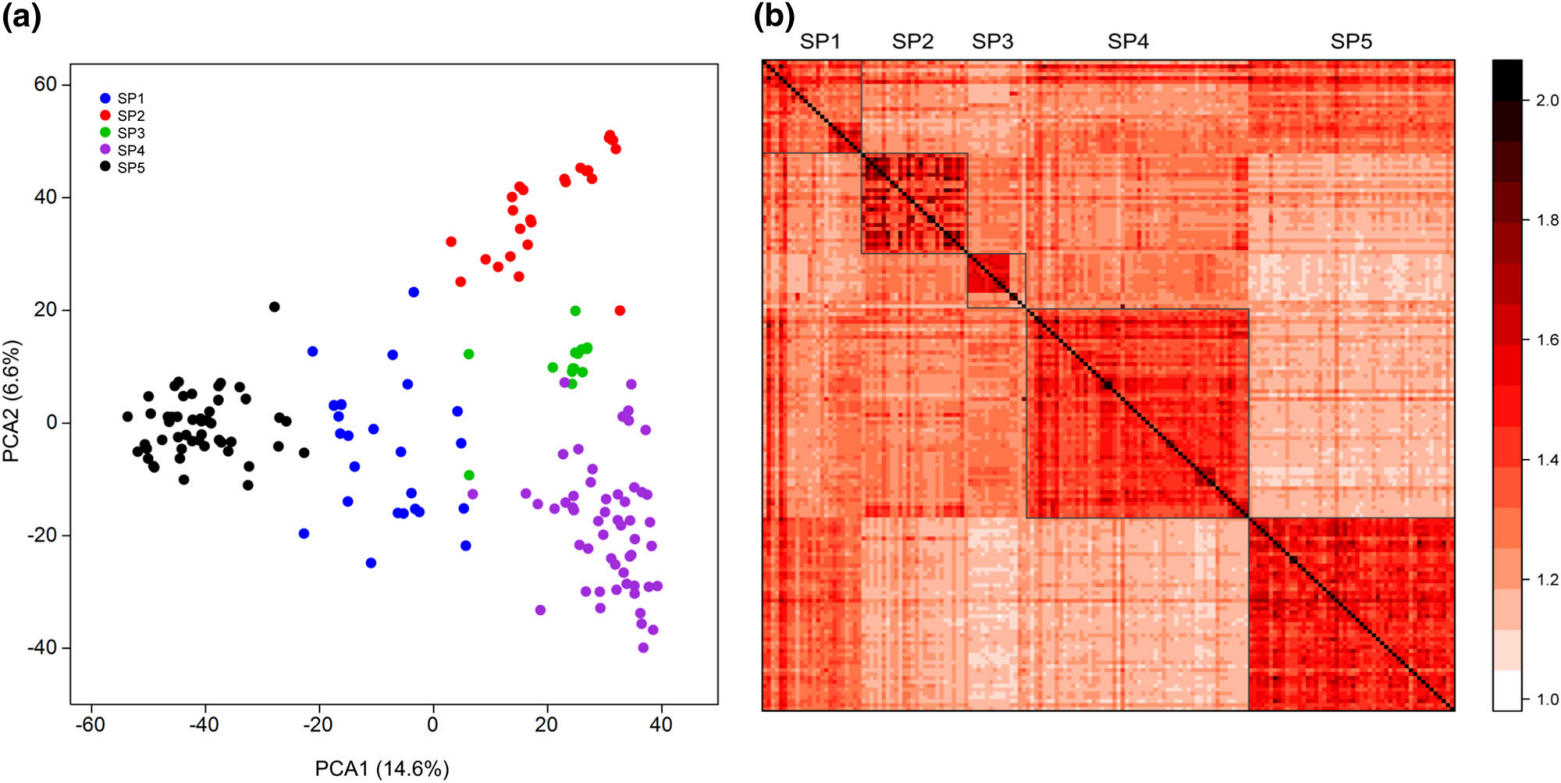
(a) Principal component analysis (PCA) of the mini‐core panel. (b) Heatmap matrix displaying the genetic kinship among the mini‐core accessions calculated from 32,942 single nucleotide polymorphism markers with corresponding subpopulations (SPs) identified by PCA

K‐means clustering separated the 160 accessions of the mini‐core panel into five subpopulations, as was previously reported for the larger iCore population (Munoz‐Amatriain et al., 2013). This was congruent with the grouping revealed by the PCA. The PCA plot showed that Principal Component 1 explained 14.6% of the variability and Principal Component 2 explained 6.6% of the variability (Figure 5a). Furthermore, the genetic similarity based on kinship analyses was consistent with the PCA and revealed an especially close genetic relationship among accessions within Subpopulations 2, 4, and 5 (Figure 5b).

Distinct geographic distributions were identified for the subpopulations on the basis of the K‐means, PCA, and genetic kinship analyses. According to the PCA plot, it appears that Subpopulation 1 accessions are equidistant and were mostly from the United States. Accessions in the Subpopulation 2 cluster were mostly from Asian countries. The smallest group was Subpopulation 3 and contained mostly accessions from Southern Europe and North African countries. The Subpopulation 4 cluster contained accessions from the Americas. All Canadian accessions, several accessions from the USA, and some South American accessions were in the Subpopulation 4 cluster. Most of the accessions in Subpopulation 5 were from Europe and South American countries.

### 3.4 Genome‐wide association study for tocols in the WBDC and mini‐core panels

In order to identify significant MTAs, a liberal criterion of the bottom 0.1 percentile for the distribution of *p*‐values has been suggested (Chan, Rowe, & Kliebenstein, 2010; Pasam et al., 2012; Zhang et al., 2017). In our study, we implemented a more stringent cutoff by using the lowest 0.0001 percentile of the *p*‐values’ distribution. In the WBDC panel, 53 QVEs were identified (Supplemental Table S3). Thirteen of these markers were associated with more than one trait, thus bringing the number of unique QVEs to 40. Haplotype analysis surrounding these significant markers indicated there were 22 haplotype blocks. Only the most significant marker within each block was selected for further analysis (Supplemental Table S4).

When we used a highly stringent three‐tiered approach for identifying MTAs, more than one significant marker could be identified for each of the tocols that also could account for 10% or more of the phenotypic variance (*R^2^*) (Table 4).

**TABLE 4:** Significant marker–trait associations in the mini‐core and Wild Barley Diversity Collection (WBDC) panels and their proximity to genes in the tocochromanol pathway in the barley genome

QVE^a^	SNP^b^	Position	Trait(s)^a^, ^c^	*P*‐value	*R* ^2^	Gene name	Abbreviaiton	Stable gene ID	Start^d^	Distance^e^
		bp							bp	
1.1	*JHI‐Hv50k‐2016‐7394*	6,332,253	BT3	2.33 × 10^–4^	0.13	*Tyrosine aminotransferase*	*TAT*	*HORVU1Hr1G015090*	43,087,960	36,755,706
1.2	S1H_31689162	31,689,162	BT	1.07 × 10^–4^	0.10	*Tyrosine aminotransferase*	*TAT*	*HORVU1Hr1G015090*	43,087,960	11,398,798
1.5	S1H_504493781	504,493,781	DT3	1.07 × 10^–6^	0.15	*Tyrosine aminotransferase*	*TAT*	*HORVU1Hr1G071260*	492,627,283	11,844,480
						*Tocopherol‐binding protein*	*TBP*	*HORVU1Hr1G075150*	509,976,502	5,482,721
1.6	S1H_531647892	531,647,892	TT	2.76 × 10^–5^	0.05	*1‐Deoxy‐d‐xylulose‐5‐phosphate synthase*	*DXS*	*HORVU1Hr1G084900*	534,540,795	2,914,921
1.7	S1H_535903897	535,903,897	DT	2.20 × 10^–6^	0.09	*1‐Deoxy‐d‐xylulose‐5‐phosphate synthase*	*DXS*	*HORVU1Hr1G084900*	534,543,485	1,360,412
2.5	*JHI‐Hv50k‐2016‐129534*	727,209,729	DT	5.54 × 10^–8^	0.15	*Homogentisate phytyltransferase*	*VTE2*	*HORVU2Hr1G117600*	740,363,384	13,153,655
						*Phytol kinase*	*VTE5*	*HORVU2Hr1G121250*	751,131,734	23,922,005
						*1‐Deoxy‐d‐xylulose‐5‐phosphate synthase*	*DXS*	*HORVU2Hr1G126150*	762,827,285	35,617,556
2.6	*JHI‐Hv50k‐2016‐135562*	742,067,194	GT3	4.71 × 10^–5^	0.17	*Homogentisate phytyltransferase*	*VTE2*	*HORVU2Hr1G117600*	740,363,384	1,703,810
						*Phytol kinase*	*VTE5*	*HORVU2Hr1G121250*	751,131,734	9,064,540
						*1‐Deoxy‐d‐xylulose‐5‐phosphate synthase*	*DXS*	*HORVU2Hr1G126150*	762,827,285	20,760,091
3.3	*JHI‐Hv50k‐2016‐183011*	487,378,898	BT	3.59 × 10^–6^	0.19	*Farensyl diphosphate synthase*	*FDPS*	*HORVU3Hr1G065960*	502,806,110	15,427,712
3.4	*JHI‐Hv50k‐2016‐185988*	512,359,049	DT3	4.37 × 10^–5^	0.17	*Phytyl‐phosphate kinase*	*VTE6*	*HORVU3Hr1G069090*	524,266,661	11,907,612
						*Prenyltransferase*	*PRT*	*HORVU3Hr1G070050*	530,325,159	17,966,110
3.5	S3H_624411648	624,411,648	BT3	1.16 × 10^–4^	0.12	*Phytol or farnesol kinase*	*VTE5*	*HORVU3Hr1G087680*	621,983,643	2,428,005
3.6	*JHI‐Hv50k‐2016‐205136*	633,067,610	AT	5.51 × 10^–5^	0.15	*Phytol or farnesol kinase*	*VTE5*	*HORVU3Hr1G087680*	621,983,643	11,080,159
						*2‐c‐Methyl‐d‐erythritol 4‐phosphate cytidylyltransferase*	*MCT*	*HORVU3Hr1G090560*	630,250,577	2,817,033
3.7	*JHI‐Hv50k‐2016‐207829*	648,559,522	DT	2.97 × 10^–7^	0.16	*2‐Methyl‐6‐phytyl‐1,4‐hydroquinone methyltransferase*	*VTE3*	*HORVU3Hr1G113670*	688,859,441	40,299,919
4.1	S4H_6536060	6,536,060	AT	1.75 × 10^–6^	0.07	*2‐Methyl‐6‐phytyl‐1,4‐hydroquinone methyltransferase*	*VTE3*	*HORVU4Hr1G002050*	3,623,194	2,912,866
4.2	S4H_591201688	591,201,688	AT	4.75 × 10^–9^	0.14	*3‐Dehydroquinolate synthase*	*DHQS*	*HORVU4Hr1G075140*	596,618,325	5,416,637
5.1	S5H_32887010	32,887,010	DT3	1.21 × 10^–5^	0.05	*2‐Methyl‐6‐phytyl‐1,4‐hydroquinone methyltransferase*	*VTE3*	*HORVU5Hr1G008980*	20,433,243	12,453,767
5.2	S5H_580322285	580,322,285	GT3	5.51 × 10^–6^	0.10	*Phytoene synthase*	*PHS*	*HORVU5Hr1G088130*	580,830,535	508,250
5.3	S5H_581775308	581,775,308	BT	8.39 × 10^–6^	0.04	*Phytoene synthase*	*PHS*	*HORVU5Hr1G088130*	580,830,535	944,773
5.4	*JHI‐Hv50k‐2016‐328186*	583,138,291	BT	1.56 × 10^–6^	0.10	*StayGreen*	*SG*	*HORVU5Hr1G081500*	564,845,583	18,292,708
						*3‐Dehydroquinolate synthase*	*DHQS*	*HORVU5Hr1G084070*	571,039,602	12,095,000
						*Phytoene synthase*	*PHS*	*HORVU5Hr1G088130*	580,830,535	2,307,756
5.5	*JHI‐Hv50k‐2016‐351248*	640,363,180	AT	2.04 × 10^–4^	0.10	*Tocopherol cyclase*	*VTE1*	*HORVU5Hr1G106680*	624,785,636	15,577,544
						*3‐Dehydroquinolate synthase*	*DHQS*	*HORVU5Hr1G109300*	629,522,666	10833696
						*Geranylgeranyl diphosphate synthase*	*GGDS*	*HORVU5Hr1G122440*	662,137,831	21,774,651
6.2	S6H_479264279	479,264,279	AT	9.95 × 10^–8^	0.09	*γ‐Tocopherol methyltransferase*	*VTE4*	*HORVU6Hr1G068410*	474,017,733	5,246,546
6.3	S6H_483619550	483,619,550	DT	1.60 × 10^–5^	0.06	*γ‐Tocopherol methyltransferase*	*VTE4*	*HORVU6Hr1G068410*	474,017,733	9,601,817
6.4	*JHI‐Hv50k‐2016‐415781*	534,653,101	DT	1.36 × 10^–5^	0.13	*Geranylgeranyl diphosphate reductase*	*GGDR*	*HORVU6Hr1G075900*	522,651,685	12,001,416
						*Tyrosine aminotransferase*	*TAT*	*HORVU6Hr1G081670*	546,165,126	11,512,025
6.5	*JHI‐Hv50k‐2016‐423804*	561,617,836	BT3	4.17 × 10^–5^	0.11	*Geranylgeranyl diphosphate reductase*	*GGDR*	*HORVU6Hr1G075900*	522,651,685	38,966,151
						*Tyrosine aminotransferase*	*TAT*	*HORVU6Hr1G081670*	546,165,126	15,452,710
6.6	*JHI‐Hv50k‐2016‐424630*	563,562,045	DT3	1.72 × 10^–4^	0.03	*Geranylgeranyl diphosphate reductase*	*GGDR*	*HORVU6Hr1G075900*	522,651,685	40,910,360
						*Tyrosine aminotransferase*	*TAT*	*HORVU6Hr1G081670*	546,165,126	17,396,919
7.1	S7H_23774978	23,774,978	DT3	9.43 × 10^–8^	0.11	*5‐Enoylpyruvylshikimate‐3‐phosphate synthase*	*EPSPS*	*HORVU7Hr1G013710*	19,653,248	4,121,730
7.2	*JHI‐Hv50k‐2016‐507055*	632,355,974	GT, TT	3.45 × 10^–8^	0.19	*Homogentisate phytyltransferase*	*VTE2*	*HORVU7Hr1G110990*	632,202,536	153,438
						*Homogentisate geranylgeranyl transferase*	*HGGT*	*HORVU7Hr1G114330*	639,390,985	7,035,011
7.3	S7H_639417725	639,417,725	AT3,BT3, GT3,TT3, TT	5.76 × 10^–7^	0.08	*Homogentisate phytyltransferase*	*VTE2*	*HORVU7Hr1G110990*	632,202,536	7,066,464
						*Homogentisate geranylgeranyl transferase*	*HGGT*	*HORVU7Hr1G114330*	639,390,985	121,985
	*JHI‐Hv50k‐2016‐510759*	639,323,737	AT3,GT3, TTC, TT	4.96 × 10^–8^	0.24	*Homogentisate phytyltransferase*	*VTE2*	*HORVU7Hr1G110990*	632,202,536	7,121,201
						*Homogentisate geranylgeranyl transferase*	*HGGT*	*HORVU7Hr1G114330*	639,390,985	67,248
7.4	S7H_652553899	652,553,899	DT	6.68 × 10^–6^	0.13	*Phytoene synthase*	*PHS*	*HORVU7Hr1G120660*	652,117,208	436,611

^a^ QVE, quantitative Vitamin E locus; TT, total tocopherol; TT3, total tocotrienol; AT, α‐tocopherol; AT3, α‐tocotrienol; BT, β‐tocopherol; BT3, β‐tocotrienol; GT, γ‐tocopherol; GT3, γ‐tocotrienol; DT, δ‐tocopherol; DT3, δ‐tocotrienol.

^b^ Single nucleotide polymorphism (SNP) markers in italics indicate these identified in the mini‐core population; regular font represents the markers in the WBDC panel.

^c^ For SNPs associated with multiple traits, only the highest *R* ^2^ and corresponding *P*‐values are provided.

^d^ Start refers to the starting position of the gene.

^e^ Distance indicates the number of bp between the SNP and the starting coordinates of the gene.

**FIGURE 6:**
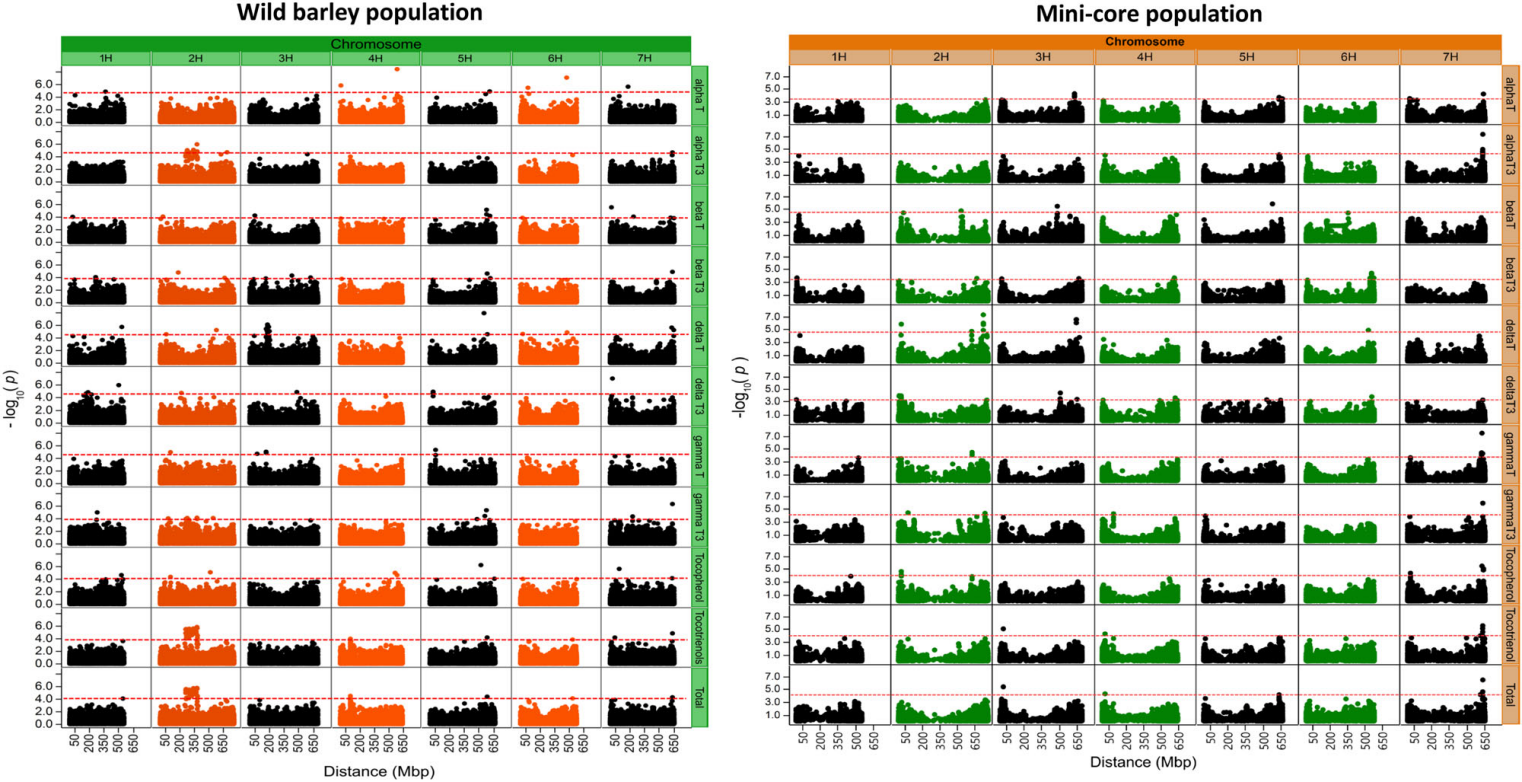
Manhattan plots displaying single nucleotide polymorphism (SNP) markers significantly associated with eight isoforms of tocols, total tocopherols, total tocotrienols, and total tocochromanols in the (a) Wild Barley Diversity Collection (WBDC) and (b) the mini‐core panel. The threshold for declaring significant SNPs for each trait using the combined model is shown as a horizontal dashed red line

Significant marker associations with the tocol traits were identified on all seven chromosomes (Figure 6). The largest number of significant SNPs (12) appeared on chromosome 7. Chromosome 2 had 11, and chromosome 5 had 10. Chromosomes 3 and 4 each had five significant SNPs; chromosome 6, with four significant SNPs, had the least. Nearly 77% of the variance in the amount of DT in the WBDC panel was accounted for by the seven significant SNP associations identified on six different chromosomes. More than 50% of the variation in the quantity of BT3 and AT was explained by five and six significant SNPs respectively. Five significant markers each for BT, DT3, and GT3 accounted for 30 to 40% of the phenotypic variation in these traits. The lowest amount of phenotypic variation explained was for the quantity of AT3, for which the significant SNPs on chromosomes 2 and 7 accounted for 17%. The marker S3H_168655778 on chromosome 3 explained 22 and 18% of the phenotypic variation in the quantity of DT and GT respectively. The marker S7H_639471725 on chromosome 7 was associated with AT3, BT3, and GT3, accounting for 7, 4, and 8%, respectively, of the variation for these traits (Supplemental Table S3).

In the mini‐core panel, 34 significant SNP markers were identified that were associated with the tocol traits (Supplemental Table S3). Seven of these markers were associated with more than one trait, thus bringing the number of unique markers to 27. Haplotype analysis surrounding these significant markers indicated there were 15 haplotype blocks. Similar to the case of the wild barley panel, significant MTAs with the tocol traits were identified on all the seven chromosomes for the mini‐core panel (Figure 6). The highest number of significant SNPs (nine) was identified on chromosomes 2 and 7. Chromosome 3 had six significant SNPs, whereas chromosomes 4, 6, 5, and 1 harbored 4, 3, 2, and 1 SNPs, respectively. Nearly 57% of the phenotypic variation in DT levels in the mini‐core panel was explained by markers on chromosomes 2, 3, and 6. Two markers, one at the proximal and other at the distal end of chromosome 2, accounted for more than 14% each of the variation in DT. Only one marker (JHI‐Hv50k‐2016‐510759 on chromosome 7) was identified as being significantly associated with AT3 and accounted for 23.5% of the variation in this isoform of Vitamin E. This marker was also found to be associated with GT3 and explained 21.5% of the variation in the total tocotrienol levels in the mini‐core panel (Supplemental Table S3).

### 3.5 Candidate genes

**FIGURE 7:**
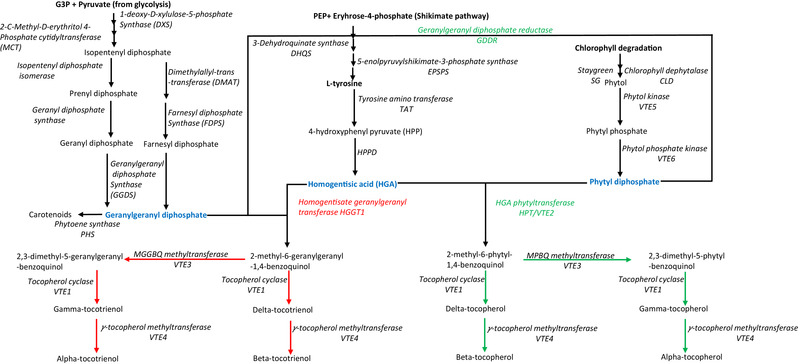
Biochemical pathway of tocochromanol biosynthesis in barley. *VTE*, Vitamin E gene; G3P, glycerol‐3‐phosphate; PEP, phospho‐enol‐pyruvate; MGGBQ, 2‐methyl‐6‐geranylgernayl‐1,4‐benzoquinol; MPBQ, 2‐methyl‐6‐phytyl‐1,4‐benzoquinol

**FIGURE 8:**
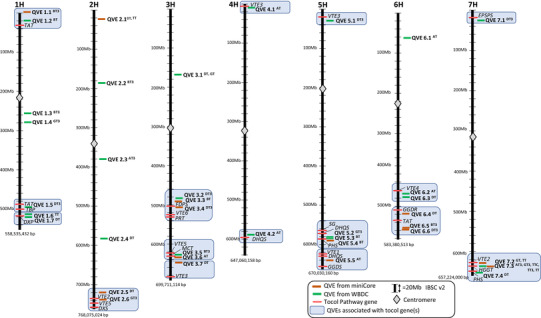
Physical map of barley chromosomes showing the location of the single nucleotide polymorphisms (SNPs) associated with vitamin E isoforms (quantitative Vitamin E loci, QVEs) identified in this study. WBDC, Wild Barley Diversity Collection; TAT, tyrosine amino transferase; TBP, tocopherol‐binding protein; DXP, 1‐deoxy‐*d*‐xylulose‐5‐phosphate synthase; FDPS, farensyl diphosphate synthase; PRT, prenyltransferase; MCT, 2‐*c*‐methyl‐*d*‐erythritol 4‐phosphate cytidylyltransferase; DHQS, 3‐dehydroquinolate synthase; SG, StayGreen; PHS, phytoene synthase; GGDS, geranylgeranyl diphosphate synthase; GGDR, geranylgeranyl diphosphate reductase; EPSPS, 5‐enoylpyruvylshikimate‐3‐phosphate synthase; HGGT, homogentysyl geranylgeranyl transferase

All the genes associated with the central tocol biosynthesis pathway (*VTE1–6*) in plants have been identified (Munne‐Bosch & Alegre, 2002) (Figure 7). The coordinates of each of these genes were retrieved from the barley database and cross‐referenced against the location of the significant SNP markers from this analysis (Table 4). Coordinates for the genes associated with the methylerythritol phosphate (MEP) and shikimate pathways were retrieved from the barley Ensembl database with the enzyme names used as keywords and were cross‐referenced to the location of the significant SNPs identified in this study. On the basis of the LD decay pattern for the mini‐core population (Supplemental Figure S2), genes that were up to 45 Mb from the SNP were included. On the other hand, the rapid decay of LD in the WBDC panel (Sallam et al., 2017) indicated the inclusion of genes within a distance of 12 Mb from the SNP. All the genes in the primary tocol pathway were in the proximity of at least one SNP from our analysis and, in several instances, the mapping revealed clusters of significant SNPs in close proximity to pathway genes (Figure 8).

There are three main precursors for this pathway: homogentisic acid, phytyl diphosphate, and GGDP. Homogentisic acid, which forms the aromatic head group of all tocols, requires tyrosine that is usually produced via the shikimate pathway (Figure 7). Significant SNPs associated with two key enzymes involved in the tyrosine biosynthetic pathway were identified. Two SNPs in the mini‐core population (JHI‐Hv50k‐2016‐328186 and JHI‐Hv50k‐2016‐351248) were 12 and 10.8 Mb from a 3‐dehydroquinolate synthase at the distal end of chromosome 5. A SNP marker (S4H_591201688) in the WBDC panel that explained 14% of the variation in AT content was 5.4 Mb from another 3‐dehydroquinolate synthase gene. A SNP marker (S7H_23774978) on chromosome 7 that accounted for 11% of the variation in DT3 in the WBDC panel was 4 Mb from the key penultimate enzyme, 5‐enoylpyruvyl‐shikimate‐3‐phosphate synthase in the tyrosine pathway. The conversion of tyrosine to homogentisic acid involves two enzymes: tyrosine amino transferase (TAT) and hydroxyphenyl pyruvate dioxygenase (Figure 7). Four SNPs significantly associated with tocols were identified in proximity to three different genes coding for TAT. A SNP in the mini‐core panel (JHI‐Hv50k‐2016‐7394) accounting for 13% of the variation in BT3 and a SNP from the WBDC panel (S13H_31689162) accounting for 10% of the variation in BT were 36 and 11.3 Mb, respectively, from a TAT gene on the proximal end of chromosome 1. In the WBDC panel, a SNP (S1H_504493781) that accounted for 15% of the variation in DT3 on the distal end of chromosome 3 was less than 12 Mb from another TAT gene (Table 4, Figure 8). A SNP (JHI‐Hv50k‐2016‐415781) identified in the mini‐core panel that explained 13% of the variation in DT mapped to the bottom of chromosome 6 was 11.5 Mb from the third TAT gene. Only a single gene coding for the hydroxyphenyl pyruvate dioxygenase was identified in the barley genome on chromosome 6. A significant SNP for AT was identified in the WBDC panel, which, however, was located ∼30 Mb from this SNP.

Phytyldiphosphate, the precursor for the hydrophobic tail group for tocopherol biosynthesis, is derived from the chlorophyll breakdown pathway. A SNP marker on chromosome 5 (JHI‐Hv50k‐2016‐328186) that accounted for 10% of the variation in BT was mapped close to a *StayGreen* gene. The latter steps in the biosynthesis of phytol diphosphate include phytol kinase (*VTE5*) and phytyl phosphate kinase (*VTE6*) (Figure 8). Two markers on chromosome 2 identified in the mini‐core panel were significantly associated with DT and GT3 and were 9 and 24 Mb, respectively, from the *VTE5* gene (Figure 8). Another *VTE5* gene was found in proximity to two markers on chromosome 3. A marker from the mini‐core population (JHI‐Hv50k‐2016‐205136) accounted for 15% of the variation in AT content and was 11 Mb from *VTE5*, whereas the marker S3H_624411648 in the WBDC panel that explained 12% variation in BT3 was 2.4 Mb from the *VTE5* gene (Table 4, Figure 8). A marker on chromosome 3 (JHI‐Hv50k‐2016‐185988) that accounted for 17% of the variation in DT3 was less than 12 Mb from the *VTE6* gene. The identification of significant SNPs for tocols on chromosomes 2 and 3 and their proximity to key enzymes for the biosynthesis of tocols has not been reported in other barley QTL studies of Vitamin E.

Geranylgeranyl diphosphate is the precursor for the hydrophobic tail group for tocotrienol biosynthesis and is derived from the plastid‐localized MEP pathway. The rate‐limiting enzyme of the MEP pathway in plants is 1‐deoxy‐*d*‐xylulose‐5‐phosphate synthase (DXP) (Estevez, Cantero, Reindl, Reichler, & Leon, 2001). Two *DXP* genes in the barley genome were identified that were in proximity to significant SNPs associated with tocols in both the wild barley and mini‐core panels. Two adjacent SNPs on chromosome 1 belonging to two different haplotype blocks in wild barley were 1.36 and 2.91 Mb from the *DXP* gene (Table 4, Figure 8). In the mini‐core panel, a SNP on chromosome 2 (JHI‐Hv50k‐2016‐135562) was ∼20 Mb from a *DXP* gene (Figure 8, Table 4). A SNP on chromosome 3 (JHI‐Hv50k‐2016‐205136) accounting for 15% of the variation in AT was 2.8 Mb from the 2‐*c*‐methyl‐*d*‐erythritol 4‐phosphate cytidylyltransferase, the third enzyme in the MEP pathway (Phillips, Leon, Boronat, & Rodriguez‐Concepcion, 2008). A SNP in the mini‐core population (JHI‐Hv50k‐2016‐1585988) that explained 17% of the variation in DT3 content was ∼18 Mb from a prenyltransferase enzyme that leads to the generation of farnesyl diphosphate. Adjacent to this marker, another SNP in the mini‐core population (JHI‐Hv50k‐2016‐183011) that accounted for 19% of the variation in BT was found to be 15 Mb from a farnesyl diphosphate synthase, which is a key enzyme for the generation of GGDP. On the distal end of chromosome 5, a significant SNP (JHI‐Hv50k‐2016‐351248) in the mini‐core population that explained 10% of the variation in AT was ∼21 Mb from a geranylgeranyl diphosphate synthase gene. Geranylgeranyl diphosphate can be converted into phytyl diphosphate by the enzyme geranylgeranyl diphosphate reductase (GGDR), thus increasing the pool size for tocopherol biosynthesis. In the mini‐core panel, a significant SNP on chromosome 6 was identified 12 Mb from a gene annotated as GGDR (Table 4, Figure 8)

The most significant MTA explaining 23.5% of the variation in the amount of AT3 in the mini‐core panel was attributed to a SNP (JHI‐Hv50k‐2016‐510759) on chromosome 7. In the WBDC panel, a marker (S7H_639471725) was identified in the same vicinity and was significantly associated with several tocotrienols (Table 4). These two markers are only 67 kb (in the mini‐core panel) and 122 kb (in the WBDC panel) from homogentysyl geranylgeranyl transferase (HGGT), the key enzyme for tocotrienol biosynthesis (Yang et al., 2011). A second marker (JHI‐Hv50k‐2016‐507055) in the mini‐core population accounted for 19% of the variation in GT and 14% for total tocopherols, and was 153 kb from homogentisate phytyltransferase, a key enzyme in the tocopherol biosynthetic pathway (Collakova & DellaPenna, 2001). Thus, a high‐density SNP array in conjunction with a large and diverse panel facilitated the identification of two discrete QVEs that are associated with two key enzymes involved in tocopherol and tocotrienol biosynthesis.

The *VTE3* gene that encodes a methyltransferase uses the products of the reactions catalyzed by HGGT and homogentisate phytyltransferase to generate dimethyl‐geranylgeranyl benzoquinols and dimethyl‐phytyl benzoquinols. Three SNPs significantly associated with tocols were found in proximity to the *VTE3* genes localized on three different chromosomes. The SNP markers on the proximal end of chromosomes 4 (S4H_6536060) and 5 (S5H_32887010) that were significantly associated with the variation in the amount of AT and DT3 in the WBDC panel were 6 and 12 Mb, respectively, from the *VTE3* locus. The SNP (JHI‐Hv50k‐2016‐207829) on the distal end of chromosome 3 in the mini‐core panel was ∼40 Mb from a *VTE3* (Table 4, Figure 8).

The *VTE1* gene that encodes a tocopherol cyclase can use the byproducts of the HGGT and homogentisate phytyltransferase enzymes or the byproducts of *VTE3* to produce either δ or γ tocols (Sattler, Cahoon, Coughlan, & DellaPenna, 2003). In barley, the amount of γ tocols is much greater than that of the δ isoforms (Table 2). A preference for the 2,3‐dimethyl‐geranylgeranyl or phytyl‐benzoquinol products generated by the VTE3 enzyme maybe one of the possible factors for the higher levels of γ tocols. A SNP (JHI‐Hv50k‐2016‐351248) in the mini‐core panel that explained 10% of the variation in AT was 15.6 Mb from a *VTE1* gene (Figure 8).

The γ‐tocopherol methyltransferase (TMT) enzyme encoded by the *VTE4* gene is the last enzyme of the pathway that converts the γ or δ isoforms into α or β isoforms, respectively. In the WBDC panel, two significant SNPs on chromosome 6 associated with AT (S6H_479264279) and DT (S6H_483619550) were 5 and 9.6 Mb, respectively, from a *VTE4* gene (Table 4, Figure 8).

We also identified eight SNPs in regions of the genome that were not identified in previous studies. Seven SNPs in the WBDC panel and one in the mini‐core panel that accounted for >10% each of the variation in the various seed tocol isoforms (Figure 8, Supplemental Table S5). For the SNP from the mini‐core population, the 45‐Mb region surrounding the SNP retrieved more than 820 proteins. In the 12‐Mb region surrounding these seven SNPs in the WBDC panel, more than 700 protein coding genes were retrieved. A number of proteins with kinase domains, methyl transferase domains, and various types of transcription factors were identified. However, domain‐based annotation did not aid in precisely identifying any specific enzymes associated with the tocol pathway.

## 4 DISCUSSION

### 4.1 Phenotypic variation for tocols

Vitamin E plays an important role in regulating lipid peroxidation, which aids in quelling oxidative damage during seed dormancy. This is supported by genetic mutants and transgenic lines with reduced Vitamin E exhibiting a significant reduction in their seed longevity (Chen, Li, Fang, Shi, & Chen, 2016; Sattler et al., 2004). In general, high germination rates in seeds are of paramount importance for farmers. Particular for barley, for which a major use of the seeds is in the malting industry, germination represents a pivotal aspect of this process. Furthermore, levels of Vitamin E in processed malt were significantly correlated with their levels in unprocessed seeds (Do, Cozzolino, Muhlhausler, Box, & Able, 2015) and can be useful for supporting the viability of brewing yeasts during the fermentation process, which, in turn is beneficial for producing higher levels of ethanol (Zhang, Qin, Lu, Wan, & Zhu, 2016). Thus examining the genetic diversity of barley germplasm to identify accessions with high levels of tocols is important for developing varieties with good malting traits.

The fact that the tocochromonal levels in processed malt are similar to the levels in unprocessed seeds (Do et al., 2015) suggests that these metabolites are very stable. This was also supported by our observation that the tocol profile of seeds from the WBDC panel collected almost a decade apart were significantly correlated (Figure 2). Furthermore, the high heritability values for each of the tocol isoforms observed in both the WBDC and mini‐core populations (Table 1) indicate that the phenotypic variation for tocol traits in barley is mostly under genetic control and can be harnessed in breeding programs.

To gain an appreciation for this vast diversity in the amount of seed tocols within these panels, one line with the highest or lowest concentrations was identified for each of the eight tocol isoforms, tocopherols, tocotrienols, and tocols (Table 2). The total tocols in the WBDC panel were more abundant (*p* < .05) than in the mini‐core panel. It can be speculated that the wild barley species that have prolonged dormancy may require higher levels of tocols to keep their seeds viable. On the same grounds, cultivated barley, represented by the mini‐core panel have been selected over time for reduced dormancy levels, which could account for their lower tocol levels. The mean tocotrienol concentrations were almost identical between these two diverse panels. However, interesting differences could be discovered from examination of the individual isoforms (Figure 2, Figure 3, Figure 4, Table 2). The mean total tocopherol level in the WBDC population was more than 40% higher than the mean of the mini‐core panel. This pattern was explained by the ∼50% higher levels of the AT isoform in the WBDC panel than in the mini‐core panel. The levels of GT3 in the two panels were <2 μg apart, with the mini‐core panel being at the higher end. Interestingly, the mean AT3 levels showed a difference of ∼4 μg between the two panels, but higher levels of this metabolite were found in the WBDC panel. It has been reported that in mature barley grains, 20% of the total tocopherol pool and 40% of the total tocotrienol pool are comprised of their respective γ‐tocochromanol isoforms (Schuy et al., 2019). In our analysis, we found that 50% of the total tocopherol and total tocotrienol pools in both the WBDC and mini‐core panels comprised the AT and AT3 forms, respectively. This pattern of distribution has also been observed in several other studies in barley (Do et al., 2015; Graebner et al., 2015; Panfili, Fratianni, & Irano, 2003). In light of our study and the abovementioned reports, it can be suggested that the activity of γ‐TMT, the final enzyme in the biosynthetic pathway of the α isoform, may not be a limiting factor in barley seeds and that, consequently, α isoforms dominate the Vitamin E profile in barley seeds.

Interestingly, the tocopherol concentrations showed a significant difference between the WBDC and mini‐core panels. Though the average GT levels were nearly identical in these two diverse panels, the AT isoform, which is the ultimate product of this pathway, had nearly twice the concentration in the WBDC panel than in the mini‐core panel. This suggests that the γ‐TMT enzyme, which is important for the conversion from the γ to the α isoform in *H. vulgare* ssp. *spontaneum* is more efficient than in *H. vulgare* ssp. *vulgare*. This merits further detailed investigation and could be harnessed for increasing the levels of tocopherols in the seeds of cultivated barley.

The δ isoform was the least abundant of the eight forms of Vitamin E in barley. The average concentration of the DT isoform was ∼30% higher in the WBDC panel than in the mini‐core panel, whereas the pattern was reversed for the DT3 isoform, which showed a mean value that was ∼50% higher in the mini‐core panel than in the WBDC panel (Table 2). The δ isoforms are the penultimate pathway products and thus could determine the amount of β isoforms. Average BT levels in the WBDC and mini‐core panels were 50% higher than DT levels, whereas the levels of BT3 were nearly fourfold higher than those of DT3, which was consistent in both panels. On the basis of these observations, it can be speculated that enhancing the metabolic flux at the initial point in the pathway may not cause any bottlenecks in the conversion of δ to β isoforms in barley seeds.

### 4.2 Population structure in the mini‐core panel

The mini‐core panel is a subset of the USDA‐ARS Barley Core Collection (*N* = 2417) and captures most of its allelic diversity (Munoz‐Amatriain et al., 2013). In the 2013 study using the 7,842 SNPs for genotyping, five major subpopulations within the core panel were reported. In our study using the 50k SNP genotyping platform, we identified five subpopulations within the mini‐core panel supported by PCA. The same population stratification was also observed, based on the genetic kinship analysis of the lines in the mini‐core panel (Figure 5). Consistent with these observations, the population structure was associated with the geographic distribution of the accessions. Accessions from Asia (Subpopulation 2) were clearly separated from the accessions from Americas (Subpopulation 4) and those from Europe and South America (Subpopulation 5). These results are in line with other reports demonstrating a strong relationship between population structure and geography in barley (Russell et al., 2016; Sallam et al., 2017).

### 4.3 Linkage disequilibrium

The LD estimates for the mini‐core panel were about fivefold higher than those reported for the WBDC panel (Table 3, Supplemental Figure S2) (Sallam et al., 2017). In the previous study of the iCore population, great variability in the LD distribution was observed between the subpopulations, as well as significant differences in the distribution of LD across the seven barley chromosomes (Munoz‐Amatriain et al., 2013). Other studies have reported that LD extends over a long range in cultivated barley and a very short range in wild barley but is intermediate in landraces (Caldwell, Russell, Langridge, & Powell, 2006; Morrell, Toleno, Lundy, & Clegg, 2005; Sallam, Endelman, Jannink, & Smith, 2015). The *H. vulgare* ssp. *spontaneum* has much lower LD than the *H. vulgare* ssp. *vulgare* and is comparable with outcrossing species like maize. This may be caused by an accumulation of outcrossing events, given the long evolution of this species, the higher frequency of chiasmata formation in inbreeding species, and the relatively recent shift in fertilization from an outcrossing to a selfing system (Morrell et al., 2005). Given this significantly different distribution of LD between the two panels used for the GWAS of tocols, significant MTAs were evaluated that took this aspect into consideration.

### 4.4 Candidate genes

Three studies of the QTLs associated with tocols in barley have been reported (Graebner et al., 2015; Oliver et al., 2014; Schuy et al., 2019). All three studies indicated that the distal region of chromosome 7 harbors a major QTL associated with tocols in barley. Consistent with these reports, in the current study, one shared segment was identified, on the basis of haplotype block analysis, in both populations that was associated with the *HGGT* gene, a key enzyme in the tocotrienol biosynthesis, and the *VTE2* gene that is vital for tocopherol biosynthesis (Figure 7, Figure 8). The *QVE7.4* locus located distally on the long arm of chromosome 7 explains 13% of the variation in DT. In the vicinity of this QVE, phytoene synthase, the first committed enzyme of the carotenoid pathway that leads to the production of phytoene by combining two molecules of GGDP, was identified (Chamovitz, Misawa, Sandmann, & Hirschberg, 1992). It is interesting to note that the closely located *QVE7.2* and *QVE7.3* loci, both in proximity to *VTE2* and the rate‐limiting HGGT enzyme for tocotrienol biosynthesis, are in the same region of the genome. Enzymes that can channel the metabolite fluxes towards the production of tocopherols and carotenoids in close proximity provide a simple and efficient mechanism to respond rapidly to changes in external cues and/or internal developmental programs (Nutzmann, Huang, & Osbourn, 2016), as has been reported in carrot (*Daucus carota* L. var. *sativus*) (Koch & Goldman, 2005; Nutzmann et al., 2016).

Two QTLs on chromosome 6 associated with tocols that were separated by ∼3 Mb have been reported earlier (Graebner et al., 2015; Oliver et al., 2014). In our GWAS analysis, we speculate that we have identified the same regions on chromosome 6 that are in proximity to the *GGDR* and *TAT* genes (Figure 7, Figure 8). These slightly discordant positions may be attributed to the differences observed between the individual genome builds used in each SNP mapping study.

In the current study, a cluster of significant SNPs mapped to the distal region of chromosome 1 in the WBDC panel whose positions overlapped two QTLs from another study that were mapped adjacent to each other (Figure 8) (Oliver et al., 2014). In the WBDC panel, SNPs in proximity to *TAT* and *DXP* were identified, supporting the possibility that this chromosome region harbors a significant source of genetic material to modulate tocols in barley seeds. A significant marker associated with BT was also identified on chromosome 1 in a survey of 1536 spring barley lines from 10 different breeding populations (Graebner et al., 2015). On the basis of their reported coordinates, we speculate it may be associated with the same *TAT* gene identified in our analysis. Interestingly, a gene with cellular retinaldehyde‐binding domain, a triple functional domain (CRAL‐TRIO) and Golgi dynamics domain was identified in the same region (*QVE1.5*)(Figure 8). Recently, the identification of the first tocopherol‐binding protein (TBP) in plants was reported in tomato (*Solanum lycopersicum* L.) (Bermudez et al., 2018) contained the CRAL‐TRIO–Golgi dynamics domain. The CRAL‐TRIO domain is also found in mammalian AT‐binding protein (Sato et al., 1993; Zimmer et al., 2000). Analysis of the barley protein sequence by ChloroP (http://www.cbs.dtu.dk/services/ChloroP/, accessed 5 Aug. 2020) indicated this protein had a chloroplast transit peptide (73 amino acids; score = 0.504) and showed 89% similarity to the tomato TBP and 74% homology to the human TBP (Supplemental Figure S3). The identification of TBP in plants suggests tocopherols play a role in inter‐ and/or intraorganellar communication (Munoz & Munne‐Bosch, 2019), which warrants further investigation.

Apart from the previously reported QTLs associated with tocols on chromosomes 7, 6, and 1, our analysis provides the first insights on chromosomes 2, 3, 4, and 5 that are associated with the precursors of tocol biosynthesis (Figure 7). The enzyme catalyzing the breakdown of chlorophyll in seed tissues to provide the phytyl moiety is still unknown (Pellaud & Mene‐Saffrane, 2017; Zhang et al., 2014). The identification of a SNP marker on chromosome 5 in proximity to the *StayGreen* is noteworthy. Interestingly, the expression of this gene was higher in the inflorescence rachis, lemma, and palea tissues than in senescing leaves (Supplemental Figure S4) suggesting this gene may play a role in providing the phytyl moiety for tocol biosynthesis in seeds, which warrants further investigation. On chromosomes 3 and 5, MTAs in both the WBDC and mini‐core panels were found in proximity to 2‐*c*‐methyl‐*d*‐erythritol 4‐phosphate cytidylyltransferase and 3‐dehydroquinolate synthase, two upstream enzymes involved in the MEP and tyrosine pathways, respectively (Figure 8). The MTAs identified at the distal end of chromosome 4 and the proximal end of chromosome 7, respectively, were found to be in close proximity to *geranylgeranyl diphosphate synthase* and *ESPS* genes that encode two enzymes that are important for the generation of the tocol precursors GGDP and tyrosine. Though these observations do not necessarily mean that these enzymes underlie these SNP–trait associations, consideration of the important pathways for the generation of precursor metabolites helped provide a biochemical context for several of the novel MTAs identified in this study.

## AUTHOR CONTRIBUTIONS

R.M and J.G.W oversaw the tocol analyses. B.J.S provided the seeds of the WBDC panel. B.J.S and A.H.S provided the genotype‐by‐sequencing data for the WBDC panel and conducted the GWAS analysis for both populations in this study. J.D.F oversaw the SNP genotyping of the mini‐core population and submission of the marker data to the T3 database. R.M and J.G.W cowrote the manuscript and all authors reviewed it.

## CONFLICT OF INTEREST STATEMENT

The authors declare that there is no conflict of interest.

## Supporting information

Supplementary Material

Supplemental Figure S1: Chromatographic separation of the eight tocol standards.

Supplemental Figure S2. Linkage disequilibrium estimated as *r* ^2^ with a sliding window of 50 adjacent markers plotted against the physical distance.

Supplemental Figure S3. Protein sequence alignment of the tomato TBP, the putative barley TBP, and the human tocopherol binding proteins made by the TCOFFEE program.

Supplemental Figure S4. Expression profile of the *StayGreen* gene retrieved from the Barlex database.

Supplemental Table S1. Tocochromanol concentrations in seeds of the WBDC panel

Supplemental Table S2. Tocochromanol concentrations in seeds of the mini‐core panel.

Supplemental Table S3. List of marker–trait associations for the WBDC and mini‐core panels

Supplemental Table S4. Haplotype blocks of significant MTAs in the WBDC and mini‐core panels

Supplemental Table S5. Gene identifiers and their domain annotations in the 45‐ and 12‐Mb regions surrounding the novel QVEs in the mini‐core and WBDC panels.

Supplemental File S1. A detailed protocol used for the determination of tocols in barley seeds.

## Data Availability

All the marker data and phenotypic trait data associated with the tocol isoforms in the mini‐core and WBDC panels are available at the T3 database (https://triticeaetoolbox.org/barley/, accessed 5 Aug. 2020).
