# Supplementary material for: Genome‐wide association analysis of natural variation in seed tocochromanols of barley
Source: Plant Genome. 2020 Sep 28;13(3):e20039. doi: 10.1002/tpg2.20039 (PMC12807016; doi:10.1002/tpg2.20039)
Supplement: Supplementary file 1 [file TPG2-13-e20039-s004.docx]

Table S3. List of marker trait associations for the WBDC and mini-core panel

| **Trait** | **SNP marker id** | **Chrom** | **Position** | **p-value** | **FDR** | ***R^2^*** |
| --- | --- | --- | --- | --- | --- | --- |
| alphaT | S4H_591059841 | 4H | 591,059,841 | 0.000000005 | 3.625E-08 | 0.14 |
| alphaT | S6H_479247818 | 6H | 479,247,818 | 0.000000099 | 5.742E-07 | 0.09 |
| alphaT | S4H_6536060 | 4H | 6,536,060 | 0.000001749 | 7.24586E-06 | 0.07 |
| alphaT | S7H_183557616 | 7H | 183,557,616 | 0.000002682 | 9.33336E-06 | 0.08 |
| alphaT | S6H_77953588 | 6H | 77,953,588 | 0.000003966 | 1.23229E-05 | 0.05 |
| alphaT | S5H_613386872 | 5H | 613,386,872 | 0.000014806 | 2.90097E-05 | 0.07 |
| alphaT | JHI-Hv50k-2016-205136 | 3H | 633,067,610 | 0.00006 | 7.29036E-05 | 0.15 |
| alphaT | JHI-Hv50k-2016-513371 | 7H | 644,457,516 | 0.00006 | 7.29036E-05 | 0.05 |
| alphaT | JHI-Hv50k-2016-351248 | 5H | 640,363,180 | 0.0002 | 0.000202326 | 0.1 |
| alphaT3 | JHI-Hv50k-2016-510759 | 7H | 639,323,737 | 0 | 0 | 0.24 |
| alphaT3 | S2H_383673613 | 2H | 383,673,613 | 0.000001062 | 4.88574E-06 | 0.1 |
| alphaT3 | S7H_639471725 | 7H | 639,471,725 | 0.000021625 | 3.61803E-05 | 0.07 |
| beta T | S7H_13265841 | 7H | 13,265,841 | 0.000003369 | 1.08557E-05 | 0.07 |
| beta T | S5H_581737422 | 5H | 581,737,422 | 0.000008393 | 2.14762E-05 | 0.04 |
| beta T | S3H_52183144 | 3H | 52,183,144 | 0.000066423 | 7.70507E-05 | 0.05 |
| beta T | S2H_29564303 | 2H | 29,564,303 | 0.000098263 | 0.000111024 | 0.05 |
| beta T | S1H_31689162 | 1H | 31,689,162 | 0.000107483 | 0.000116888 | 0.1 |
| beta T3 | S7H_639471725 | 7H | 639,471,725 | 0.000015005 | 2.90097E-05 | 0.04 |
| beta T3 | S2H_189421020 | 2H | 189,421,020 | 0.000019274 | 3.41176E-05 | 0.12 |
| beta T3 | S5H_587439038 | 5H | 587,439,038 | 0.000028397 | 4.41168E-05 | 0.06 |
| beta T3 | S1H_267358715 | 1H | 267,358,715 | 0.000101362 | 0.000111836 | 0.16 |
| beta T3 | S3H_624368913 | 3H | 624,368,913 | 0.000116216 | 0.000123302 | 0.12 |
| betaT | JHI-Hv50k-2016-183011 | **3H** | 487,378,898 | 0 | 0 | 0.19 |
| betaT | JHI-Hv50k-2016-328186 | 5H | 583,138,291 | 0 | 0 | 0.1 |
| betaT | JHI-Hv50k-2016-97027 | 2H | 537,377,447 | 0.00002 | 3.41176E-05 | 0.04 |
| betaT3 | JHI-Hv50k-2016-423804 | 6H | 561,617,836 | 0.00004 | 5.70492E-05 | 0.11 |
| betaT3 | JHI-Hv50k-2016-7394 | 1H | 6,332,253 | 0.0002 | 0.000202326 | 0.13 |
| betaT3 | JHI-Hv50k-2016-264420 | 4H | 617,796,694 | 0.00023 | 0.00023 | 0.03 |
| delta T | S5H_555105922 | 5H | 555,105,922 | 0.000000015 | 1.00385E-07 | 0.09 |
| delta T | S1H_535881879 | 1H | 535,881,879 | 0.000002201 | 7.97863E-06 | 0.09 |
| delta T | S3H_168655778 | 3H | 168,655,778 | 0.000004443 | 0.000013329 | 0.22 |
| delta T | S7H_652553899 | 7H | 652,553,899 | 0.00000668 | 1.78719E-05 | 0.13 |
| delta T | S2H_585177594 | 2H | 585,177,594 | 0.000006779 | 1.78719E-05 | 0.12 |
| delta T | S6H_483603089 | 6H | 483,603,089 | 0.000015975 | 2.95707E-05 | 0.06 |
| delta T | S6H_23941078 | 6H | 23,941,078 | 0.000027476 | 4.36265E-05 | 0.06 |
| delta T3 | S7H_23774978 | 7H | 23,774,978 | 0.000000094 | 5.742E-07 | 0.11 |
| delta T3 | S1H_504471763 | 1H | 504,471,763 | 0.000001067 | 4.88574E-06 | 0.15 |
| delta T3 | S5H_32887010 | 5H | 32,887,010 | 0.000012135 | 2.70701E-05 | 0.05 |
| delta T3 | S3H_483614803 | 3H | 483,614,803 | 0.000013363 | 2.83556E-05 | 0.1 |
| delta T3 | S2H_222161497 | 2H | 222,161,497 | 0.000018497 | 3.35258E-05 | 0.05 |
| deltaT | JHI-Hv50k-2016-129534 | 2H | 727,209,729 | 0 | 0 | 0.15 |
| deltaT | JHI-Hv50k-2016-207829 | 3H | 648,559,522 | 0 | 0 | 0.16 |
| deltaT | JHI-Hv50k-2016-71482 | **2H** | 23,209,276 | 0.000002 | 7.7203E-06 | 0.14 |
| deltaT | JHI-Hv50k-2016-415781 | 6H | 534,653,101 | 0.000014 | 2.88617E-05 | 0.13 |
| deltaT3 | JHI-Hv50k-2016-185988 | 3H | 512,359,049 | 0.00004 | 5.70492E-05 | 0.17 |
| deltaT3 | JHI-Hv50k-2016-61924 | 2H | 5,924,708 | 0.00011 | 0.000118148 | 0 |
| deltaT3 | JHI-Hv50k-2016-70801 | 2H | 21,821,052 | 0.00012 | 0.000125783 | 0.05 |
| deltaT3 | JHI-Hv50k-2016-424630 | 6H | 563,562,045 | 0.00017 | 0.000176071 | 0.03 |
| gamma T | S5H_58347099 | 5H | 58,347,099 | 0.000005443 | 1.54523E-05 | 0.04 |
| gamma T | S2H_107643680 | 2H | 107,643,680 | 0.000012446 | 2.70701E-05 | 0.03 |
| gamma T | S3H_168655778 | 3H | 168,655,778 | 0.000015543 | 2.93965E-05 | 0.18 |
| gamma T3 | S7H_639471725 | 7H | 639,471,725 | 0.000000576 | 0.000003132 | 0.08 |
| gamma T3 | S5H_580284399 | 5H | 580,284,399 | 0.000005506 | 1.54523E-05 | 0.1 |
| gamma T3 | S1H_283412443 | 1H | 283,412,443 | 0.00001202 | 2.70701E-05 | 0.1 |
| gamma T3 | S7H_230802092 | 7H | 230,802,092 | 0.000052971 | 6.77717E-05 | 0.05 |
| gamma T3 | S2H_383673613 | 2H | 383,673,613 | 0.00008324 | 9.52879E-05 | 0.07 |
| gammaT | JHI-Hv50k-2016-507055 | 7H | 632,355,974 | 0 | 0 | 0.19 |
| gammaT | JHI-Hv50k-2016-103586 | 2H | 631,136,690 | 0.00003 | 4.57895E-05 | 0.08 |
| gammaT3 | JHI-Hv50k-2016-510759 | 7H | 639,323,737 | 0 | 0 | 0.07 |
| gammaT3 | JHI-Hv50k-2016-82292 | 2H | 80,834,384 | 0.00004 | 5.70492E-05 | 0.01 |
| gammaT3 | JHI-Hv50k-2016-135562 | 2H | 742,067,194 | 0.00005 | 6.59091E-05 | 0.17 |
| gammaT3 | JHI-Hv50k-2016-239647 | 4H | 95,778,191 | 0.00006 | 7.29036E-05 | 0 |
| Tocopherol | JHI-Hv50k-2016-507055 | 7H | 632,355,974 | 0 | 0 | 0.14 |
| Tocopherol | S5H_523483934 | 5H | 523,483,934 | 0.000000728 | 3.72565E-06 | 0.08 |
| Tocopherol | S7H_93033412 | 7H | 93,033,412 | 0.000002887 | 9.66035E-06 | 0.06 |
| Tocopherol | S2H_521977254 | 2H | 521,977,254 | 0.000009987 | 2.35135E-05 | 0.05 |
| Tocopherol | JHI-Hv50k-2016-513371 | 7H | 644,457,516 | 0.00001 | 2.35135E-05 | 0.07 |
| Tocopherol | JHI-Hv50k-2016-71533 | **2H** | 23,217,764 | 0.00002 | 3.41176E-05 | 0.12 |
| Tocopherol | S4H_591059841 | 4H | 591,059,841 | 0.000027551 | 4.36265E-05 | 0.05 |
| Tocopherol | S1H_531625874 | 1H | 531,625,874 | 0.00002758 | 4.36265E-05 | 0.05 |
| Tocopherol | JHI-Hv50k-2016-453148 | 7H | 24,061,095 | 0.00005 | 6.59091E-05 | 0.09 |
| Tocopherol | S2H_107643680 | 2H | 107,643,680 | 0.00005196 | 6.74704E-05 | 0.04 |
| Tocotrienol | JHI-Hv50k-2016-510759 | 7H | 639,323,737 | 0 | 0 | 0.21 |
| Tocotrienol | JHI-Hv50k-2016-161081 | 3H | 28,157,704 | 0.00001 | 2.35135E-05 | 0.06 |
| Tocotrienol | JHI-Hv50k-2016-232096 | 4H | 23,375,449 | 0.00005 | 6.59091E-05 | 0.04 |
| Tocotrienols | S2H_383673613 | 2H | 383,673,613 | 0.000001367 | 5.94645E-06 | 0.08 |
| Tocotrienols | S7H_639269000 | 7H | 639,269,000 | 0.000014265 | 2.88617E-05 | 0.06 |
| Tocotrienols | S5H_587439038 | 5H | 587,439,038 | 0.000064752 | 7.68045E-05 | 0.06 |
| Tocotrienols | S7H_48191089 | 7H | 48,191,089 | 0.000065328 | 7.68045E-05 | 0.06 |
| Tocotrienols | S4H_105267236 | 4H | 105,267,236 | 0.000101552 | 0.000111836 | 0.04 |
| Total | JHI-Hv50k-2016-161081 | 3H | 28,157,704 | 0 | 0 | 0.07 |
| Total | JHI-Hv50k-2016-510759 | 7H | 639,323,737 | 0 | 0 | 0.15 |
| Total | S2H_383673613 | 2H | 383,673,613 | 0.000002041 | 7.7203E-06 | 0.08 |
| Total | S4H_105267236 | 4H | 105,267,236 | 0.00003612 | 0.00005418 | 0.05 |
| Total | S5H_587439038 | 5H | 587,439,038 | 0.000047812 | 6.59091E-05 | 0.06 |
| Total | JHI-Hv50k-2016-232096 | 4H | 23,375,449 | 0.00005 | 6.59091E-05 | 0.03 |
| Total | S7H_639269000 | 7H | 639,269,000 | 0.000060334 | 7.29036E-05 | 0.05 |
