# Supplementary material for: Genome‐wide association analysis of natural variation in seed tocochromanols of barley
Source: Plant Genome. 2020 Sep 28;13(3):e20039. doi: 10.1002/tpg2.20039 (PMC12807016; doi:10.1002/tpg2.20039)
Supplement: Supplementary file 2 [file TPG2-13-e20039-s007.docx]

*Modified procedure for tocochromanol analysis of barley seeds*

Briefly, 100 mg freshly-ground sample was weighed into a 5-mL Sarstadt tube and mixed with 0.25 mL potassium hydroxide (600 g/L H_2_O), 0.25 mL ethanol (95%), 0.25 mL sodium chloride (10 g/L H_2_O), and 0.62 mL ethanolic pyrogallol (60 g/L EtOH). Tubes were then vortexed and saponified in a 70 ^o^C water bath for 30 minutes. Tubes were chilled in an ice bath for at least 30 minutes. The solution was mixed with 1.8 mL sodium chloride (10 g/L) and 1.8 mL n-hexane/ethyl acetate (9:1), vortexed and centrifuged at 3000 rpm for seven minutes. The top layer was transferred with a glass pipette and mixed with 1.8 mL n-hexane/ethyl acetate, vortexed and centrifuged at 3000 rpm for seven minutes. The top layer was transferred to new tubes and the organic layers were evaporated in a speed vacuum (Thermo Savant) at 45 ^o^C for one hour.

Dried residue was resuspended in 0.25 mL hexane. Chromatographic separation was performed with normal phase HPLC, using a Shimadzu (Kyoto, Japan) LC-6A pump, RF10AxL detector, and SCL-10Avp controller. Compounds were separated on a 5-mm 25064.6 mm Grace Adsorbosil silica column using an isocratic mobile phase (hexane with 2% dioxane and 2% ethyl acetate) at a flow rate of 2 mL/min. Peaks were detected by fluorescence with excitation at 295 nm and emission at 330 nm.
