# Supplementary material for: Genome‐wide association analysis of natural variation in seed tocochromanols of barley
Source: Plant Genome. 2020 Sep 28;13(3):e20039. doi: 10.1002/tpg2.20039 (PMC12807016; doi:10.1002/tpg2.20039)
Supplement: Supplementary file 3 [file TPG2-13-e20039-s010.docx]

| **Table S5. Gene identifiers and their domain annotations in the 12 Mb or 45 Mb regions** | | | |
| --- | --- | --- | --- |
|  |  | **Gene stable ID** | **Interpro Short Description** |
| **Population** | **WBDC** | HORVU1Hr1G038060 | Amidohydro-rel |
| **Segment** | **SEGMENT-03** | HORVU1Hr1G038070 | Small_GTPase |
| **Haplotype** | **Haplo-02** | HORVU1Hr1G038130 | Znf_RING |
| **QVE ID** | **QVE1.3** | HORVU1Hr1G038180 | AAA+_ATPase |
| **SNP** | **S1H_267358715** | HORVU1Hr1G038220 | Pec_lyase |
| **Chromosome** | **1** | HORVU1Hr1G038240 | RRM_dom |
| **SNP position** | **267,358,715** | HORVU1Hr1G038260 | Shugoshin_C |
| **Allele** | **G/A** | HORVU1Hr1G038270 | Ham1p-like |
| **Upstream** | **261358715** | HORVU1Hr1G038280 | Peptidase_S1_PA |
| **Downstream** | **273358715** | HORVU1Hr1G038320 | Tubby-like_C |
|  |  | HORVU1Hr1G038330 | Thiolase |
|  |  | HORVU1Hr1G038370 | SNF2_N |
|  |  | HORVU1Hr1G038380 | Znf_CCHC_sf |
|  |  | HORVU1Hr1G038500 | Exostosin |
|  |  | HORVU1Hr1G038510 | Exostosin |
|  |  | HORVU1Hr1G038560 | Leu-rich_rpt |
|  |  | HORVU1Hr1G038620 | SAP_dom |
|  |  | HORVU1Hr1G038650 | BTB/POZ_dom |
|  |  | HORVU1Hr1G038670 | Sec23/24_trunk_dom |
|  |  | HORVU1Hr1G038680 | Chaprnin_Cpn60 |
|  |  | HORVU1Hr1G038700 | tRNAsynth_Ia_anticodon-bd |
|  |  | HORVU1Hr1G038730 | GDSL |
|  |  | HORVU1Hr1G038740 | UspA |
|  |  | HORVU1Hr1G038810 | Band_7 |
|  |  | HORVU1Hr1G038820 | WD40_repeat |
|  |  | HORVU1Hr1G038850 | Riboflavin_kinase_bac/euk |
|  |  | HORVU1Hr1G038870 | Znf_RING |
|  |  | HORVU1Hr1G038880 | WD40_repeat |
|  |  | HORVU1Hr1G038890 | Ribosomal_L21e |
|  |  | HORVU1Hr1G038920 | Glyco_hydro_14 |
|  |  | HORVU1Hr1G038950 | Glyco_hydro_14 |
|  |  | HORVU1Hr1G038990 | PfkB_dom |
|  |  | HORVU1Hr1G039010 | P-loop_NTPase |
|  |  | HORVU1Hr1G039030 | Ribosomal_L11/L12 |
|  |  | HORVU1Hr1G039050 | Ubox_domain |
|  |  | HORVU1Hr1G039090 | Mediator_Med18 |
|  |  | HORVU1Hr1G039140 | BTB/POZ_dom |
|  |  |  |  |
| **Population** | **WBDC** | HORVU1Hr1G039500 | Prot_kinase_dom |
| **Segment** | **SEGMENT-04** | HORVU1Hr1G039540 | DAGAT |
| **Haplotype** | **Haplo-03** | HORVU1Hr1G039570 |  |
| **QVE ID** | **QVE1.4** | HORVU1Hr1G039600 | Leu-rich_rpt |
| **SNP** | **S1H_283412443** | HORVU1Hr1G039610 | ND/Mrp_mem |
| **Chromosome** | **1** | HORVU1Hr1G039620 | KH_dom-like_a/b |
| **SNP position** | **283,412,443** | HORVU1Hr1G039630 | ATP_synth_F1_dsu/esu |
| **Allele** | **C/T** | HORVU1Hr1G039670 | POT_fam |
| **Upstream** | **277412443** | HORVU1Hr1G039720 | POT_fam |
| **Downstream** | **289412443** | HORVU1Hr1G039760 | PS_antenna-like |
|  |  | HORVU1Hr1G039790 | RRM_dom |
|  |  | HORVU1Hr1G039810 | unc-13_homologue |
|  |  | HORVU1Hr1G039820 | Fatty_acid_hydroxylase |
|  |  | HORVU1Hr1G039830 | Fatty_acid_hydroxylase |
|  |  | HORVU1Hr1G039870 | Cyanate_lyase_C |
|  |  | HORVU1Hr1G039880 | Helicase_C |
|  |  | HORVU1Hr1G040050 |  |
|  |  | HORVU1Hr1G040060 |  |
|  |  | HORVU1Hr1G040070 | RNA_pol_Rpb1_1 |
|  |  | HORVU1Hr1G040110 | CDI |
|  |  | HORVU1Hr1G040130 |  |
|  |  | HORVU1Hr1G040140 | Bifunc_inhib/LTP/seed_store |
|  |  | HORVU1Hr1G040150 | Glia_glutenin |
|  |  | HORVU1Hr1G040160 | PPPDE_dom |
|  |  | HORVU1Hr1G040170 | aa-tRNA-synth_I_CS |
|  |  | HORVU1Hr1G040180 | PPPDE_dom |
|  |  | HORVU1Hr1G040200 | WD40_repeat |
|  |  | HORVU1Hr1G040250 | WD40_repeat |
|  |  | HORVU1Hr1G040260 | RVT-Znf |
|  |  | HORVU1Hr1G040300 |  |
|  |  | HORVU1Hr1G040310 | Znf_CCHC |
|  |  | HORVU1Hr1G040320 | RraA/RraA-like |
|  |  | HORVU1Hr1G040330 |  |
|  |  | HORVU1Hr1G040370 |  |
|  |  | HORVU1Hr1G040420 | 3-ktacl-CoA_syn |
|  |  | HORVU1Hr1G040540 |  |
|  |  | HORVU1Hr1G040560 | UDP-g_GGtrans |
|  |  | HORVU1Hr1G040620 | Peptidase_C48_C |
|  |  | HORVU1Hr1G040640 |  |
|  |  | HORVU1Hr1G040660 | GMC_OxRdtase_N |
|  |  | HORVU1Hr1G040670 | PurL_large |
|  |  | HORVU1Hr1G040700 | UbiB_dom |
|  |  | HORVU1Hr1G040720 | GMC_OxRdtase_N |
|  |  | HORVU1Hr1G040740 |  |
|  |  | HORVU1Hr1G040800 | Ubiquitin-like_dom |
|  |  | HORVU1Hr1G040810 | bHLH_dom |
|  |  | HORVU1Hr1G040850 | Prot_kinase_dom |
|  |  | HORVU1Hr1G040920 |  |
|  |  | HORVU1Hr1G040930 | Snf7_fam |
|  |  | HORVU1Hr1G040940 | Hsp_70_fam |
|  |  |  |  |
| **Population** | **WBDC** | HORVU2Hr1G038740 | MIP |
| **Segment** | **SEGMENT-09** | HORVU2Hr1G038760 | Znf_AN1 |
| **Haplotype** | **Haplo-07** | HORVU2Hr1G038770 |  |
| **QVE ID** | **QVE2.2** | HORVU2Hr1G038790 | Prot_kinase_dom |
| **SNP** | **S2H_189421020** | HORVU2Hr1G038800 | Methyltransf_16 |
| **Chromosome** | **2** | HORVU2Hr1G038820 |  |
| **SNP position** | **189,421,020** | HORVU2Hr1G038830 | OxRdtase_FAD/NAD-bd |
| **Allele** | **G/C** | HORVU2Hr1G038850 | ATPase_BadF |
| **Upstream** | **183421020** | HORVU2Hr1G038860 | WAP |
| **Downstream** | **195421020** | HORVU2Hr1G038940 | PSII_PsbR |
|  |  | HORVU2Hr1G038950 | Prot_kinase_dom |
|  |  | HORVU2Hr1G038960 | PSII_PsbR |
|  |  | HORVU2Hr1G038980 | Cyt_P450_sf |
|  |  | HORVU2Hr1G038990 | Leu-rich_rpt |
|  |  | HORVU2Hr1G039000 |  |
|  |  | HORVU2Hr1G039030 | Remorin_C |
|  |  | HORVU2Hr1G039040 |  |
|  |  | HORVU2Hr1G039050 | Cop_transporter |
|  |  | HORVU2Hr1G039100 | Tic20 |
|  |  | HORVU2Hr1G039120 | TMEM147 |
|  |  | HORVU2Hr1G039130 | Znf_PHD |
|  |  | HORVU2Hr1G039150 |  |
|  |  | HORVU2Hr1G039170 | Cullin_repeat-like_dom_sf |
|  |  | HORVU2Hr1G039180 |  |
|  |  | HORVU2Hr1G039190 |  |
|  |  | HORVU2Hr1G039210 |  |
|  |  | HORVU2Hr1G039220 |  |
|  |  | HORVU2Hr1G039250 |  |
|  |  | HORVU2Hr1G039270 |  |
|  |  | HORVU2Hr1G039300 |  |
|  |  | HORVU2Hr1G039320 |  |
|  |  | HORVU2Hr1G039340 |  |
|  |  | HORVU2Hr1G039350 |  |
|  |  | HORVU2Hr1G039360 |  |
|  |  | HORVU2Hr1G039370 |  |
|  |  | HORVU2Hr1G039390 |  |
|  |  | HORVU2Hr1G039400 |  |
|  |  | HORVU2Hr1G039500 |  |
|  |  | HORVU2Hr1G039510 | Cyt_c-like_dom_sf |
|  |  | HORVU2Hr1G039520 | Cyt_P450 |
|  |  | HORVU2Hr1G039540 |  |
|  |  | HORVU2Hr1G039570 | GlycoTrfase_DXD_sugar-bd_CS |
|  |  | HORVU2Hr1G039600 |  |
|  |  | HORVU2Hr1G039640 | NAC-dom |
|  |  | HORVU2Hr1G039660 |  |
|  |  | HORVU2Hr1G039670 | TIR_dom |
|  |  | HORVU2Hr1G039680 | FAD/NAD-bd_sf |
|  |  | HORVU2Hr1G039700 | Exostosin |
|  |  | HORVU2Hr1G039710 | PPM-type_phosphatase_dom |
|  |  | HORVU2Hr1G039850 | MATE_fam |
|  |  | HORVU2Hr1G039880 | Cyclophilin-type_PPIase_dom |
|  |  | HORVU2Hr1G039890 | AB_hydrolase_1 |
|  |  | HORVU2Hr1G039910 | Ser-Thr/Tyr_kinase_cat_dom |
|  |  | HORVU2Hr1G039930 | Put_cell_wall |
|  |  | HORVU2Hr1G039940 | Ribosomal_L5 |
|  |  | HORVU2Hr1G039960 | LRR_dom_sf |
|  |  | HORVU2Hr1G039990 | Bromodomain |
|  |  | HORVU2Hr1G040000 | DUF707 |
|  |  | HORVU2Hr1G040020 | Sec7_dom |
|  |  | HORVU2Hr1G040050 | Histone_H4 |
|  |  | HORVU2Hr1G040110 |  |
|  |  | HORVU2Hr1G040120 | Znf_CCHC |
|  |  | HORVU2Hr1G040130 | FAD/NAD-bd_sf |
|  |  | HORVU2Hr1G040140 | SCAMP |
|  |  | HORVU2Hr1G040170 | MORF/ORRM1/DAG-like |
|  |  | HORVU2Hr1G040180 | P_typ_ATPase |
|  |  | HORVU2Hr1G040270 |  |
|  |  |  |  |
| **Population** | **WBDC** | HORVU2Hr1G058220 |  |
| **Segment** | **SEGMENT-10** | HORVU2Hr1G058230 |  |
| **Haplotype** | **Haplo-08** | HORVU2Hr1G058260 | START_lipid-bd_dom |
| **QVE ID** | **QVE2.3** | HORVU2Hr1G058370 | EIF3C_N_dom |
| **SNP** | **S2H_383673613** | HORVU2Hr1G058380 | Glyco_hydro_5 |
| **Chromosome** | **2** | HORVU2Hr1G058390 | EIF3C_N_dom |
| **SNP position** | **383,673,613** | HORVU2Hr1G058530 | Prot_kinase_dom |
| **Allele** | **C/T** | HORVU2Hr1G058600 |  |
| **Upstream** | **377673613** | HORVU2Hr1G058700 | Peptidase_C19_UCH |
| **Downstream** | **389673613** | HORVU2Hr1G058720 | Ribosomal_L6 |
|  |  | HORVU2Hr1G058740 |  |
|  |  | HORVU2Hr1G058760 |  |
|  |  | HORVU2Hr1G058770 | FHY3/FAR1 |
|  |  | HORVU2Hr1G058790 |  |
|  |  | HORVU2Hr1G058800 | Cytosine_MeTrfase1_RFD |
|  |  | HORVU2Hr1G058810 |  |
|  |  | HORVU2Hr1G058820 | Prot_kinase_dom |
|  |  | HORVU2Hr1G058840 |  |
|  |  | HORVU2Hr1G058850 | Znf_CCCH |
|  |  | HORVU2Hr1G058860 | TB2_DP1_HVA22 |
|  |  | HORVU2Hr1G058900 | Prot_kinase_dom |
|  |  | HORVU2Hr1G058930 | ACP-like_sf |
|  |  | HORVU2Hr1G058940 | Hen1_Lam_C |
|  |  | HORVU2Hr1G059060 | PDZ |
|  |  | HORVU2Hr1G059090 |  |
|  |  |  |  |
| **Population** | **WBDC** | HORVU2Hr1G079960 | GGT_peptidase |
| **Segment** | **SEGMENT-11** | HORVU2Hr1G080020 | Leu-rich_rpt |
| **Haplotype** | **Haplo-09** | HORVU2Hr1G080030 | EPFL |
| **QVE ID** | **QVE2.4** | HORVU2Hr1G080040 | PS_antenna-like |
| **SNP** | **S2H_584414279** | HORVU2Hr1G080050 |  |
| **Chromosome** | **2** | HORVU2Hr1G080060 | Aldose_1/G6P_1-epimerase |
| **SNP position** | **584,414,279** | HORVU2Hr1G080070 | Aldose_1/G6P_1-epimerase |
| **Allele** | **G/T** | HORVU2Hr1G080100 | Aldose_1/G6P_1-epimerase |
| **Upstream** | **578414279** | HORVU2Hr1G080130 | Villin/Gelsolin |
| **Downstream** | **590414279** | HORVU2Hr1G080140 | Aldose_1/G6P_1-epimerase |
|  |  | HORVU2Hr1G080170 | Pectinesterase_cat |
|  |  | HORVU2Hr1G080180 |  |
|  |  | HORVU2Hr1G080190 |  |
|  |  | HORVU2Hr1G080200 | ABC_transporter-like |
|  |  | HORVU2Hr1G080210 |  |
|  |  | HORVU2Hr1G080240 |  |
|  |  | HORVU2Hr1G080260 | PSII_PsbQ |
|  |  | HORVU2Hr1G080310 | Peptidase_S1C |
|  |  | HORVU2Hr1G080320 | PPM-type_phosphatase_dom |
|  |  | HORVU2Hr1G080340 | Ribosomal_L34Ae |
|  |  | HORVU2Hr1G080350 | AA_transpt_TM |
|  |  | HORVU2Hr1G080360 | Pentatricopeptide_repeat |
|  |  | HORVU2Hr1G080370 | ATPase_ArsA/GET3 |
|  |  | HORVU2Hr1G080390 | AA_transpt_TM |
|  |  | HORVU2Hr1G080400 | At4g02000-like |
|  |  | HORVU2Hr1G080410 | At4g02000-like |
|  |  | HORVU2Hr1G080420 | AA_transpt_TM |
|  |  | HORVU2Hr1G080430 | AA_transpt_TM |
|  |  | HORVU2Hr1G080440 |  |
|  |  | HORVU2Hr1G080450 | Mopterin_CF_biosynth-C_dom |
|  |  | HORVU2Hr1G080460 | NAC-dom |
|  |  | HORVU2Hr1G080480 | Ribosomal_PSRP3/Ycf65 |
|  |  | HORVU2Hr1G080490 | TF_MADSbox |
|  |  | HORVU2Hr1G080550 | PLAC_motif_containing |
|  |  | HORVU2Hr1G080570 | Bifunc_inhib/LTP/seed_store |
|  |  | HORVU2Hr1G080580 |  |
|  |  | HORVU2Hr1G080590 | AA_transpt_TM |
|  |  | HORVU2Hr1G080630 | 14-3-3 |
|  |  | HORVU2Hr1G080720 |  |
|  |  | HORVU2Hr1G080770 | Prot_kinase_dom |
|  |  | HORVU2Hr1G080820 | GTD-bd |
|  |  | HORVU2Hr1G080860 | Pentatricopeptide_repeat |
|  |  | HORVU2Hr1G080870 | Ccc1_fam |
|  |  | HORVU2Hr1G080890 |  |
|  |  | HORVU2Hr1G080910 | Znf_RING |
|  |  | HORVU2Hr1G080940 |  |
|  |  | HORVU2Hr1G080970 | Aldehyde_DH_dom |
|  |  | HORVU2Hr1G080980 | Ribosome_inactivat_prot |
|  |  | HORVU2Hr1G080990 | Bifunc_inhib/LTP/seed_store |
|  |  | HORVU2Hr1G081010 | Ribosome_inactivat_prot |
|  |  | HORVU2Hr1G081020 | Ribosomal_L1-like |
|  |  | HORVU2Hr1G081030 | POT_fam |
|  |  | HORVU2Hr1G081040 | POT_fam |
|  |  | HORVU2Hr1G081050 | POT_fam |
|  |  | HORVU2Hr1G081060 |  |
|  |  | HORVU2Hr1G081080 | UBX_dom |
|  |  | HORVU2Hr1G081140 | RNA-binding_CRM |
|  |  | HORVU2Hr1G081150 |  |
|  |  | HORVU2Hr1G081160 |  |
|  |  | HORVU2Hr1G081180 |  |
|  |  | HORVU2Hr1G081210 | GNAT_dom |
|  |  | HORVU2Hr1G081220 | PC-Esterase |
|  |  | HORVU2Hr1G081230 | Bet_v_I/MLP |
|  |  | HORVU2Hr1G081260 | RRM_dom |
|  |  | HORVU2Hr1G081300 |  |
|  |  | HORVU2Hr1G081320 | AAA+_ATPase |
|  |  | HORVU2Hr1G081330 | PPI |
|  |  | HORVU2Hr1G081340 | Ribosomal_S19 |
|  |  | HORVU2Hr1G081360 | ARM-type_fold |
|  |  | HORVU2Hr1G081370 | Peptidyl-Arg-deiminase_porph |
|  |  | HORVU2Hr1G081380 |  |
|  |  |  |  |
| **Population** | **WBDC** | HORVU3Hr1G032120 |  |
| **Segment** | **SEGMENT-14** | HORVU3Hr1G032160 | Glyco_hydro_14 |
| **Haplotype** | **Haplo-10** | HORVU3Hr1G032170 | PB1_dom |
| **QVE ID** | **QVE3.1** | HORVU3Hr1G032200 | ABI |
| **SNP** | **S3H_168655778** | HORVU3Hr1G032230 | B3_DNA-bd |
| **Chromosome** | **3** | HORVU3Hr1G032260 | Expansin |
| **SNP position** | **168,655,778** | HORVU3Hr1G032270 | Expansin |
| **Allele** | **T/A** | HORVU3Hr1G032280 |  |
| **Upstream** | **162655778** | HORVU3Hr1G032290 | Expansin |
| **Downstream** | **174655778** | HORVU3Hr1G032300 |  |
|  |  | HORVU3Hr1G032340 | Ribosomal_S8 |
|  |  | HORVU3Hr1G032350 | CASP/CASPL |
|  |  | HORVU3Hr1G032370 | NmrA-like |
|  |  | HORVU3Hr1G032380 |  |
|  |  | HORVU3Hr1G032390 |  |
|  |  | HORVU3Hr1G032400 | Aminotrans_IV |
|  |  | HORVU3Hr1G032410 |  |
|  |  | HORVU3Hr1G032420 | Sof1 |
|  |  | HORVU3Hr1G032440 |  |
|  |  | HORVU3Hr1G032460 |  |
|  |  | HORVU3Hr1G032480 | DnaJ_domain |
|  |  | HORVU3Hr1G032490 | Tpt_PEP_transl |
|  |  | HORVU3Hr1G032530 |  |
|  |  | HORVU3Hr1G032540 | Prot_kinase_dom |
|  |  | HORVU3Hr1G032570 |  |
|  |  | HORVU3Hr1G032650 | DUF1618 |
|  |  | HORVU3Hr1G032660 | DUF1618 |
|  |  | HORVU3Hr1G032680 | NAC-dom |
|  |  | HORVU3Hr1G032690 |  |
|  |  | HORVU3Hr1G032700 |  |
|  |  | HORVU3Hr1G032710 | Longin-like_dom_sf |
|  |  | HORVU3Hr1G032730 | aa-tRNA-synth_I_CS |
|  |  | HORVU3Hr1G032740 | tRNAHis_GuaTrfase_Thg1 |
|  |  | HORVU3Hr1G032750 |  |
|  |  | HORVU3Hr1G032760 |  |
|  |  | HORVU3Hr1G032790 | Metallo-depent_PP-like |
|  |  | HORVU3Hr1G032820 | Glutaredoxin |
|  |  | HORVU3Hr1G032920 | WAP |
|  |  | HORVU3Hr1G032930 | RNaseH-like_sf |
|  |  | HORVU3Hr1G032940 | WAP |
|  |  | HORVU3Hr1G032960 | WAP |
|  |  | HORVU3Hr1G032990 | RNaseH_sf |
|  |  | HORVU3Hr1G033000 | WAP |
|  |  | HORVU3Hr1G033090 |  |
|  |  | HORVU3Hr1G033100 |  |
|  |  | HORVU3Hr1G033120 | Frigida |
|  |  | HORVU3Hr1G033140 |  |
|  |  | HORVU3Hr1G033150 |  |
|  |  | HORVU3Hr1G033160 | Pectinesterase_cat |
|  |  | HORVU3Hr1G033180 | Frigida |
|  |  | HORVU3Hr1G033190 |  |
|  |  | HORVU3Hr1G033210 |  |
|  |  | HORVU3Hr1G033220 | Cu-oxidase |
|  |  | HORVU3Hr1G033230 |  |
|  |  | HORVU3Hr1G033240 |  |
|  |  | HORVU3Hr1G033250 | Ankyrin_rpt |
|  |  | HORVU3Hr1G033290 |  |
|  |  | HORVU3Hr1G033330 |  |
|  |  |  |  |
| **Population** | **WBDC** | HORVU3Hr1G062590 | Ribosomal_L37ae |
| **Segment** | **SEGMENT-15** | HORVU3Hr1G062600 | Calycin |
| **Haplotype** | **Haplo-11** | HORVU3Hr1G062620 | YTH_domain |
| **QVE ID** | **QVE3.2** | HORVU3Hr1G062680 |  |
| **SNP** | **S3H_483657538** | HORVU3Hr1G062690 | PUP_plant |
| **Chromosome** | **3** | HORVU3Hr1G062700 | COQ10_START |
| **SNP position** | **483,657,538** | HORVU3Hr1G062710 | TAFII28 |
| **Allele** | **A/G** | HORVU3Hr1G062730 | F-box-like_dom_sf |
| **Upstream** | **477657538** | HORVU3Hr1G062740 | DUF1639 |
| **Downstream** | **489657538** | HORVU3Hr1G062760 | Transl_elong_EFG/EF2_IV |
|  |  | HORVU3Hr1G062790 |  |
|  |  | HORVU3Hr1G062800 | Harbinger_derived_prot |
|  |  | HORVU3Hr1G062830 | Harbinger_derived_prot |
|  |  | HORVU3Hr1G062860 |  |
|  |  | HORVU3Hr1G062870 | DOMON_domain |
|  |  | HORVU3Hr1G062890 |  |
|  |  | HORVU3Hr1G062900 | O-acyltransferase_WSD1_N |
|  |  | HORVU3Hr1G062920 |  |
|  |  | HORVU3Hr1G062940 | O-acyltransferase_WSD1_N |
|  |  | HORVU3Hr1G062970 | Atg6/Beclin |
|  |  | HORVU3Hr1G062980 | Sde2_N |
|  |  | HORVU3Hr1G062990 | EGF-type_Asp/Asn_hydroxyl_site |
|  |  | HORVU3Hr1G063000 | Prot_kinase_dom |
|  |  | HORVU3Hr1G063030 |  |
|  |  | HORVU3Hr1G063050 | Glu_synthdom |
|  |  | HORVU3Hr1G063060 |  |
|  |  | HORVU3Hr1G063180 |  |
|  |  | HORVU3Hr1G063220 | SF3a60_bindingd |
|  |  | HORVU3Hr1G063250 | Gaa1 |
|  |  | HORVU3Hr1G063270 | Histone_H3/CENP-A |
|  |  | HORVU3Hr1G063280 |  |
|  |  | HORVU3Hr1G063300 | AAA+_ATPase |
|  |  | HORVU3Hr1G063310 |  |
|  |  | HORVU3Hr1G063320 | ORC4 |
|  |  | HORVU3Hr1G063350 | Ribosomal_L14P |
|  |  | HORVU3Hr1G063380 |  |
|  |  | HORVU3Hr1G063430 | MATE_fam |
|  |  | HORVU3Hr1G063450 | ASF1-like |
|  |  | HORVU3Hr1G063460 | SANT/Myb |
|  |  | HORVU3Hr1G063470 | DRMBL |
|  |  | HORVU3Hr1G063500 | RVT-Znf |
|  |  | HORVU3Hr1G063560 |  |
|  |  | HORVU3Hr1G063590 | ATPase_F1/V1/A1_a/bsu_nucl-bd |
|  |  | HORVU3Hr1G063610 |  |
|  |  | HORVU3Hr1G063620 | MAP65_Ase1_PRC1 |
|  |  | HORVU3Hr1G063630 | DUF4228_plant |
|  |  | HORVU3Hr1G063640 | DUF4228_plant |
|  |  | HORVU3Hr1G063660 | UDP_glucos_trans |
|  |  | HORVU3Hr1G063670 | WD40_repeat |
|  |  | HORVU3Hr1G063680 | Znf_RING-CH |
|  |  | HORVU3Hr1G063690 | Methyltranfer_dom |
|  |  | HORVU3Hr1G063710 | Formyl_transf_N |
|  |  | HORVU3Hr1G063720 | Formyl_transf_N |
|  |  | HORVU3Hr1G063730 |  |
|  |  | HORVU3Hr1G063770 |  |
|  |  | HORVU3Hr1G063780 | SRRP53 |
|  |  | HORVU3Hr1G063790 | Formyl_trans_C |
|  |  | HORVU3Hr1G063810 |  |
|  |  | HORVU3Hr1G063840 | Hydrolase_4 |
|  |  | HORVU3Hr1G063860 | Ist1 |
|  |  | HORVU3Hr1G063960 |  |
|  |  | HORVU3Hr1G064040 | DUF1639 |
|  |  | HORVU3Hr1G064070 | DUF1639 |
|  |  | HORVU3Hr1G064080 | Znf_RING |
|  |  |  |  |
| **Population** | **WBDC** | HORVU6Hr1G022580 | Rossmann-like_a/b/a_fold |
| **Segment** | **SEGMENT-25** | HORVU6Hr1G022590 | Znf_RING |
| **Haplotype** | **Haplo-18** | HORVU6Hr1G022610 | DMP |
| **QVE ID** | **QVE6.1** | HORVU6Hr1G022660 | Asp/Asn-tRNA-synth_IIb |
| **SNP** | **S6H_77953588** | HORVU6Hr1G022670 | Znf_PMZ |
| **Chromosome** | **6** | HORVU6Hr1G022680 | LEA_2 |
| **SNP position** | **77,953,588** | HORVU6Hr1G022750 | Dirigent |
| **Allele** | **C/T** | HORVU6Hr1G022760 | TF_GRAS |
| **Upstream** | **71953588** | HORVU6Hr1G022770 | FN3_dom |
| **Downstream** | **83953588** | HORVU6Hr1G022810 |  |
|  |  | HORVU6Hr1G022890 |  |
|  |  | HORVU6Hr1G022920 |  |
|  |  | HORVU6Hr1G022950 |  |
|  |  | HORVU6Hr1G022960 | PSI_Ycf4_assembly |
|  |  | HORVU6Hr1G022970 | Ribosomal_L33 |
|  |  | HORVU6Hr1G022980 | Ribosomal_L20 |
|  |  | HORVU6Hr1G022990 | ATP_synth_F1_dsu/esu |
|  |  | HORVU6Hr1G023000 | CemA |
|  |  | HORVU6Hr1G023010 | ClpP |
|  |  | HORVU6Hr1G023040 | DUF547 |
|  |  | HORVU6Hr1G023050 | P-loop_NTPase |
|  |  | HORVU6Hr1G023070 | RF2K-like |
|  |  | HORVU6Hr1G023090 | Prot_kinase_dom |
|  |  | HORVU6Hr1G023100 | Leu-rich_rpt |
|  |  | HORVU6Hr1G023130 |  |
|  |  | HORVU6Hr1G023140 |  |
|  |  | HORVU6Hr1G023150 | NUDIX_hydrolase_dom |
|  |  | HORVU6Hr1G023180 | Initiation_fac_eIF4g_MI |
|  |  | HORVU6Hr1G023200 |  |
|  |  | HORVU6Hr1G023210 | Leu-rich_rpt |
|  |  | HORVU6Hr1G023260 | GINS_bundle-like_dom_sf |
|  |  | HORVU6Hr1G023290 | Leu-rich_rpt |
|  |  | HORVU6Hr1G023310 | GINS_bundle-like_dom_sf |
|  |  | HORVU6Hr1G023340 | Prot_kinase_dom |
|  |  | HORVU6Hr1G023350 |  |
|  |  | HORVU6Hr1G023370 | Prot_kinase_dom |
|  |  | HORVU6Hr1G023380 |  |
|  |  | HORVU6Hr1G023390 |  |
|  |  | HORVU6Hr1G023400 |  |
|  |  | HORVU6Hr1G023420 |  |
|  |  | HORVU6Hr1G023500 |  |
|  |  | HORVU6Hr1G023510 |  |
|  |  | HORVU6Hr1G023530 | Prot_kinase_dom |
|  |  | HORVU6Hr1G023550 | LRR_dom_sf |
|  |  | HORVU6Hr1G023600 |  |
|  |  | HORVU6Hr1G023620 |  |
|  |  | HORVU6Hr1G023630 | JAMM/MPN+_dom |
|  |  | HORVU6Hr1G023650 |  |
|  |  | HORVU6Hr1G023670 |  |
|  |  | HORVU6Hr1G023720 |  |
|  |  | HORVU6Hr1G023730 | Prot_kinase_dom |
|  |  | HORVU6Hr1G023740 |  |
|  |  | HORVU6Hr1G023770 | Leu-rich_rpt |
|  |  | HORVU6Hr1G023790 | NPL |
|  |  | HORVU6Hr1G023820 | DNA_glycosylase |
|  |  | HORVU6Hr1G023860 | GEBP |
|  |  | HORVU6Hr1G023870 |  |
|  |  | HORVU6Hr1G023890 |  |
|  |  | HORVU6Hr1G023980 | GEBP |
|  |  | HORVU6Hr1G024000 |  |
|  |  | HORVU6Hr1G024020 |  |
|  |  | HORVU6Hr1G024100 | Enoyl-CoA_hydra/iso |
|  |  | HORVU6Hr1G024110 |  |
|  |  | HORVU6Hr1G024120 | Znf_C2H2_type |
|  |  | HORVU6Hr1G024140 |  |
|  |  | HORVU6Hr1G024180 |  |
|  |  | HORVU6Hr1G024190 |  |
|  |  |  |  |
| **Population** | **Minicore** | HORVU2Hr1G000450 | vWFA_dom_sf |
| **Segment** | **SEGMENT-08** | HORVU2Hr1G000480 | Cyt_P450 |
| **Haplotype** | **Haplo-02** | HORVU2Hr1G000490 | Oxid_FAD_bind_N |
| **QVE ID** | **QVE2.1** | HORVU2Hr1G000510 | LRR_dom_sf |
| **SNP** | **JHI-Hv50k-2016-71482** | HORVU2Hr1G000530 | Oxid_FAD_bind_N |
| **Chromosome** | **2** | HORVU2Hr1G000540 | Oxid_FAD_bind_N |
| **SNP position** | **23,209,276** | HORVU2Hr1G000550 | BBE |
| **Allele** | **G/A** | HORVU2Hr1G000560 | NB-ARC |
| **Upstream** | **1209276** | HORVU2Hr1G000570 |  |
| **Downstream** | **45209276** | HORVU2Hr1G000580 | TRM6 |
|  |  | HORVU2Hr1G000590 | G_patch_dom |
|  |  | HORVU2Hr1G000610 | LRR_dom_sf |
|  |  | HORVU2Hr1G000620 |  |
|  |  | HORVU2Hr1G000660 | Cyt_P450 |
|  |  | HORVU2Hr1G000670 | Cyt_P450 |
|  |  | HORVU2Hr1G000700 | NB-ARC |
|  |  | HORVU2Hr1G000760 | LRR_dom_sf |
|  |  | HORVU2Hr1G000770 | NB-ARC-LRR |
|  |  | HORVU2Hr1G000780 |  |
|  |  | HORVU2Hr1G000840 | Transferase |
|  |  | HORVU2Hr1G000890 | Cyt_P450 |
|  |  | HORVU2Hr1G000900 | NB-ARC |
|  |  | HORVU2Hr1G000910 | LRR_dom_sf |
|  |  | HORVU2Hr1G000940 | SET_dom |
|  |  | HORVU2Hr1G000970 |  |
|  |  | HORVU2Hr1G001010 |  |
|  |  | HORVU2Hr1G001020 |  |
|  |  | HORVU2Hr1G001030 |  |
|  |  | HORVU2Hr1G001070 | LEA_2 |
|  |  | HORVU2Hr1G001110 | Cyt_P450 |
|  |  | HORVU2Hr1G001120 | Terpene_synth_N |
|  |  | HORVU2Hr1G001130 | Cyt_P450 |
|  |  | HORVU2Hr1G001150 | Cyt_P450 |
|  |  | HORVU2Hr1G001160 | Cyt_P450 |
|  |  | HORVU2Hr1G001180 | ABI |
|  |  | HORVU2Hr1G001240 | Dirigent |
|  |  | HORVU2Hr1G001250 | Serpin_fam |
|  |  | HORVU2Hr1G001280 | NADH_Q_OxRdtase_suD |
|  |  | HORVU2Hr1G001290 |  |
|  |  | HORVU2Hr1G001310 |  |
|  |  | HORVU2Hr1G001320 | POT_fam |
|  |  | HORVU2Hr1G001330 | Serpin_fam |
|  |  | HORVU2Hr1G001340 | P-loop_NTPase |
|  |  | HORVU2Hr1G001350 | PPIase_PpiC |
|  |  | HORVU2Hr1G001360 | tRNA_hU_synthase |
|  |  | HORVU2Hr1G001380 | POT_fam |
|  |  | HORVU2Hr1G001390 | Scorpion_toxin-like_sf |
|  |  | HORVU2Hr1G001400 | B3_DNA-bd |
|  |  | HORVU2Hr1G001410 | P-loop_NTPase |
|  |  | HORVU2Hr1G001430 |  |
|  |  | HORVU2Hr1G001530 | ABC_transporter-like |
|  |  | HORVU2Hr1G001540 | GASA |
|  |  | HORVU2Hr1G001550 | Prot_kinase_dom |
|  |  | HORVU2Hr1G001560 | Prot_kinase_dom |
|  |  | HORVU2Hr1G001600 | DH_E1 |
|  |  | HORVU2Hr1G001620 |  |
|  |  | HORVU2Hr1G001630 | ABC_transporter-like |
|  |  | HORVU2Hr1G001640 |  |
|  |  | HORVU2Hr1G001660 | Cyt_P450_sf |
|  |  | HORVU2Hr1G001680 |  |
|  |  | HORVU2Hr1G001690 | 3'-5'_exonuclease_dom |
|  |  | HORVU2Hr1G001740 |  |
|  |  | HORVU2Hr1G001750 | Agenet-like_dom |
|  |  | HORVU2Hr1G001760 | Agenet-like_dom |
|  |  | HORVU2Hr1G001770 |  |
|  |  | HORVU2Hr1G001780 |  |
|  |  | HORVU2Hr1G001790 |  |
|  |  | HORVU2Hr1G001810 | YTH_domain |
|  |  | HORVU2Hr1G001820 | Flavin_mOase |
|  |  | HORVU2Hr1G001830 | Coa1 |
|  |  | HORVU2Hr1G001870 |  |
|  |  | HORVU2Hr1G001900 | Sec23/24_trunk_dom |
|  |  | HORVU2Hr1G001960 | Prot_kinase_dom |
|  |  | HORVU2Hr1G002010 | Haem_peroxidase |
|  |  | HORVU2Hr1G002020 | Prot_kinase_dom |
|  |  | HORVU2Hr1G002040 | UDP_glucos_trans |
|  |  | HORVU2Hr1G002050 | AAR2 |
|  |  | HORVU2Hr1G002130 | Prot_kinase_dom |
|  |  | HORVU2Hr1G002160 | Thaumatin |
|  |  | HORVU2Hr1G002180 | PfkB_dom |
|  |  | HORVU2Hr1G002240 |  |
|  |  | HORVU2Hr1G002250 | Cyt_P450 |
|  |  | HORVU2Hr1G002260 | PfkB_dom |
|  |  | HORVU2Hr1G002280 | Thaumatin |
|  |  | HORVU2Hr1G002290 |  |
|  |  | HORVU2Hr1G002320 |  |
|  |  | HORVU2Hr1G002340 | GDSL |
|  |  | HORVU2Hr1G002350 | Ribosomal_L35A |
|  |  | HORVU2Hr1G002410 | Muniscin_C |
|  |  | HORVU2Hr1G002420 |  |
|  |  | HORVU2Hr1G002440 | Cu_oxidase_Cu_BS |
|  |  | HORVU2Hr1G002450 |  |
|  |  | HORVU2Hr1G002460 | Cyt_P450 |
|  |  | HORVU2Hr1G002480 | Muniscin_C |
|  |  | HORVU2Hr1G002500 | CAT-like_dom_sf |
|  |  | HORVU2Hr1G002510 |  |
|  |  | HORVU2Hr1G002520 | Cupredoxin |
|  |  | HORVU2Hr1G002570 | Cyt_P450 |
|  |  | HORVU2Hr1G002580 | Cyt_P450 |
|  |  | HORVU2Hr1G002600 | CBS_dom |
|  |  | HORVU2Hr1G002620 | AB_hydrolase_3 |
|  |  | HORVU2Hr1G002630 | AdipoR/HlyIII-related |
|  |  | HORVU2Hr1G002640 | Chalcone/stilbene_synthase_N |
|  |  | HORVU2Hr1G002650 | Cyt_P450 |
|  |  | HORVU2Hr1G002660 | Prot_kinase_dom |
|  |  | HORVU2Hr1G002690 | NB-ARC |
|  |  | HORVU2Hr1G002700 | DA1-like |
|  |  | HORVU2Hr1G002710 |  |
|  |  | HORVU2Hr1G002720 | PLAC_motif_containing |
|  |  | HORVU2Hr1G002750 | Prot_kinase_dom |
|  |  | HORVU2Hr1G002800 |  |
|  |  | HORVU2Hr1G002830 | Prot_kinase_dom |
|  |  | HORVU2Hr1G002840 | Prot_kinase_dom |
|  |  | HORVU2Hr1G002880 | Pec_lyase |
|  |  | HORVU2Hr1G002920 |  |
|  |  | HORVU2Hr1G002930 | Pec_lyase |
|  |  | HORVU2Hr1G002970 | P-loop_NTPase |
|  |  | HORVU2Hr1G003030 | P-loop_NTPase |
|  |  | HORVU2Hr1G003040 | P-loop_NTPase |
|  |  | HORVU2Hr1G003060 | P-loop_NTPase |
|  |  | HORVU2Hr1G003070 | P-loop_NTPase |
|  |  | HORVU2Hr1G003080 | P-loop_NTPase |
|  |  | HORVU2Hr1G003110 | GDA1_CD39_NTPase |
|  |  | HORVU2Hr1G003120 | P-loop_NTPase |
|  |  | HORVU2Hr1G003130 | DUF4220 |
|  |  | HORVU2Hr1G003150 | DUF594 |
|  |  | HORVU2Hr1G003180 | DUF4220 |
|  |  | HORVU2Hr1G003210 | Cyt_P450 |
|  |  | HORVU2Hr1G003220 | Znf_CCHC |
|  |  | HORVU2Hr1G003230 | P-loop_NTPase |
|  |  | HORVU2Hr1G003240 | 3'-5'_exonuclease_dom |
|  |  | HORVU2Hr1G003260 |  |
|  |  | HORVU2Hr1G003280 | Chalcone/stilbene_synthase_N |
|  |  | HORVU2Hr1G003290 | FPG_cat |
|  |  | HORVU2Hr1G003300 | AB_hydrolase_3 |
|  |  | HORVU2Hr1G003310 | WRKY_dom |
|  |  | HORVU2Hr1G003340 |  |
|  |  | HORVU2Hr1G003350 | Ribosomal_S13-like_H2TH |
|  |  | HORVU2Hr1G003360 | Terpene_synth_N |
|  |  | HORVU2Hr1G003370 | CDT1_Gemini-bd-like |
|  |  | HORVU2Hr1G003390 | Terpene_synth_N |
|  |  | HORVU2Hr1G003400 | Cyt_P450 |
|  |  | HORVU2Hr1G003420 | Terpene_synthase_metal-bd |
|  |  | HORVU2Hr1G003430 | Cyt_P450 |
|  |  | HORVU2Hr1G003450 | Cyt_P450 |
|  |  | HORVU2Hr1G003460 | ClpS_core |
|  |  | HORVU2Hr1G003470 | Cyt_P450 |
|  |  | HORVU2Hr1G003480 | Cyt_P450 |
|  |  | HORVU2Hr1G003500 | Cyt_P450 |
|  |  | HORVU2Hr1G003510 |  |
|  |  | HORVU2Hr1G003540 | Exo70 |
|  |  | HORVU2Hr1G003580 | Serpin_fam |
|  |  | HORVU2Hr1G003600 | NB-ARC |
|  |  | HORVU2Hr1G003620 |  |
|  |  | HORVU2Hr1G003630 |  |
|  |  | HORVU2Hr1G003640 | Prot_kinase_dom |
|  |  | HORVU2Hr1G003650 |  |
|  |  | HORVU2Hr1G003670 | Myb/SANT-like_dom |
|  |  | HORVU2Hr1G003680 | PLC-like_Pdiesterase_TIM-brl |
|  |  | HORVU2Hr1G003720 | GDSL |
|  |  | HORVU2Hr1G003740 | Chromo-like_dom_sf |
|  |  | HORVU2Hr1G003750 | Prot_kinase_dom |
|  |  | HORVU2Hr1G003770 | SGNH_hydro_sf |
|  |  | HORVU2Hr1G003780 | Ankyrin_rpt |
|  |  | HORVU2Hr1G003800 | NTF2 |
|  |  | HORVU2Hr1G003810 | Amidase_dom |
|  |  | HORVU2Hr1G003820 |  |
|  |  | HORVU2Hr1G003870 |  |
|  |  | HORVU2Hr1G003880 | Peptidase_S8/S53_dom |
|  |  | HORVU2Hr1G003900 | DIOX_N |
|  |  | HORVU2Hr1G003910 | Prot_kinase_dom |
|  |  | HORVU2Hr1G003920 | LRR_N_plant-typ |
|  |  | HORVU2Hr1G003940 | Oxoglu/Fe-dep_dioxygenase |
|  |  | HORVU2Hr1G003950 | Peptidase_S8/S53_dom |
|  |  | HORVU2Hr1G003970 |  |
|  |  | HORVU2Hr1G003980 | AS_sf |
|  |  | HORVU2Hr1G003990 | Amidase_dom |
|  |  | HORVU2Hr1G004000 | Cyt_P450 |
|  |  | HORVU2Hr1G004020 | AS_sf |
|  |  | HORVU2Hr1G004030 | AS_sf |
|  |  | HORVU2Hr1G004040 |  |
|  |  | HORVU2Hr1G004070 | NB-ARC |
|  |  | HORVU2Hr1G004100 |  |
|  |  | HORVU2Hr1G004130 | NAC-dom |
|  |  | HORVU2Hr1G004140 | LRR_dom_sf |
|  |  | HORVU2Hr1G004150 | NB-ARC |
|  |  | HORVU2Hr1G004170 | Chalcone/stilbene_synthase_N |
|  |  | HORVU2Hr1G004180 | Oxoglu/Fe-dep_dioxygenase |
|  |  | HORVU2Hr1G004190 | Oxoglu/Fe-dep_dioxygenase |
|  |  | HORVU2Hr1G004200 | Oxoglu/Fe-dep_dioxygenase |
|  |  | HORVU2Hr1G004230 | OxRdtase_FMN_N |
|  |  | HORVU2Hr1G004280 | DIOX_N |
|  |  | HORVU2Hr1G004290 |  |
|  |  | HORVU2Hr1G004300 | NADH_UbQ_OxRdtase_su1/FPO |
|  |  | HORVU2Hr1G004330 |  |
|  |  | HORVU2Hr1G004340 |  |
|  |  | HORVU2Hr1G004440 | Cyt_P450 |
|  |  | HORVU2Hr1G004460 | Actin |
|  |  | HORVU2Hr1G004480 | Cyt_P450 |
|  |  | HORVU2Hr1G004500 | Cyt_P450 |
|  |  | HORVU2Hr1G004510 | Cyt_P450 |
|  |  | HORVU2Hr1G004530 | Cyt_P450 |
|  |  | HORVU2Hr1G004540 | Terpene_synth_N |
|  |  | HORVU2Hr1G004550 | Cyt_P450 |
|  |  | HORVU2Hr1G004590 | Cyt_f |
|  |  | HORVU2Hr1G004600 | Cyt_P450 |
|  |  | HORVU2Hr1G004610 | Cyt_P450 |
|  |  | HORVU2Hr1G004620 | Terpene_synth_N |
|  |  | HORVU2Hr1G004640 | Cyt_P450 |
|  |  | HORVU2Hr1G004660 | DUF724 |
|  |  | HORVU2Hr1G004690 | Chalcone/stilbene_synthase_N |
|  |  | HORVU2Hr1G004710 | O_MeTrfase_dom |
|  |  | HORVU2Hr1G004720 | UDP_glucos_trans |
|  |  | HORVU2Hr1G004730 | ABC1_TM_dom |
|  |  | HORVU2Hr1G004790 | UDP_glucos_trans |
|  |  | HORVU2Hr1G004820 | E2F |
|  |  | HORVU2Hr1G004830 | Prot_kinase_dom |
|  |  | HORVU2Hr1G004850 | Serpin_fam |
|  |  | HORVU2Hr1G004870 |  |
|  |  | HORVU2Hr1G004890 | Rossmann-like_a/b/a_fold |
|  |  | HORVU2Hr1G004900 | Serpin_fam |
|  |  | HORVU2Hr1G004920 |  |
|  |  | HORVU2Hr1G004930 |  |
|  |  | HORVU2Hr1G004960 | Nicotian_synth |
|  |  | HORVU2Hr1G004980 |  |
|  |  | HORVU2Hr1G005000 | Dienelactn_hydro |
|  |  | HORVU2Hr1G005020 |  |
|  |  | HORVU2Hr1G005040 | Serpin_fam |
|  |  | HORVU2Hr1G005140 | D-isomer_DH_NAD-bd |
|  |  | HORVU2Hr1G005150 |  |
|  |  | HORVU2Hr1G005160 | DUF4378 |
|  |  | HORVU2Hr1G005210 |  |
|  |  | HORVU2Hr1G005220 | Chalcone/stilbene_synthase_N |
|  |  | HORVU2Hr1G005230 | D-isomer_2_OHA_DH_cat_dom |
|  |  | HORVU2Hr1G005310 | CAT-like_dom_sf |
|  |  | HORVU2Hr1G005320 | Ureohydrolase |
|  |  | HORVU2Hr1G005330 |  |
|  |  | HORVU2Hr1G005340 | LRR_dom_sf |
|  |  | HORVU2Hr1G005350 | Transferase |
|  |  | HORVU2Hr1G005420 | Thiolase-like |
|  |  | HORVU2Hr1G005430 |  |
|  |  | HORVU2Hr1G005470 | MFS |
|  |  | HORVU2Hr1G005480 | Adenylat/UMP-CMP_kin |
|  |  | HORVU2Hr1G005490 | Serpin_fam |
|  |  | HORVU2Hr1G005510 | Dimeric_a/b-barrel |
|  |  | HORVU2Hr1G005520 | Dimeric_a/b-barrel |
|  |  | HORVU2Hr1G005530 |  |
|  |  | HORVU2Hr1G005550 | Peptidase_S8/S53_dom |
|  |  | HORVU2Hr1G005560 | Peptidase_S8/S53_dom |
|  |  | HORVU2Hr1G005570 | Oxoglu/Fe-dep_dioxygenase |
|  |  | HORVU2Hr1G005590 | Dimeric_a/b-barrel |
|  |  | HORVU2Hr1G005600 |  |
|  |  | HORVU2Hr1G005620 |  |
|  |  | HORVU2Hr1G005630 |  |
|  |  | HORVU2Hr1G005640 |  |
|  |  | HORVU2Hr1G005650 | ARM-like |
|  |  | HORVU2Hr1G005660 | Oxid_FAD_bind_N |
|  |  | HORVU2Hr1G005670 | Oxoglu/Fe-dep_dioxygenase |
|  |  | HORVU2Hr1G005680 | DUF1295 |
|  |  | HORVU2Hr1G005690 | U5_MeTrfase_fam |
|  |  | HORVU2Hr1G005760 | Ankyrin_rpt |
|  |  | HORVU2Hr1G005790 |  |
|  |  | HORVU2Hr1G005810 |  |
|  |  | HORVU2Hr1G005820 |  |
|  |  | HORVU2Hr1G005830 |  |
|  |  | HORVU2Hr1G005870 | DUF4220 |
|  |  | HORVU2Hr1G005880 | DUF594 |
|  |  | HORVU2Hr1G005910 | DUF594 |
|  |  | HORVU2Hr1G005940 | Znf_TAZ |
|  |  | HORVU2Hr1G005980 |  |
|  |  | HORVU2Hr1G005990 | Nucl-diP-sugar_transferase |
|  |  | HORVU2Hr1G006000 | Glyco_trans_43 |
|  |  | HORVU2Hr1G006010 | PCI_dom |
|  |  | HORVU2Hr1G006030 | TMEM115/Pdh1/Rbl19 |
|  |  | HORVU2Hr1G006040 | NB-ARC |
|  |  | HORVU2Hr1G006070 |  |
|  |  | HORVU2Hr1G006090 | Jacalin-like_lectin_dom |
|  |  | HORVU2Hr1G006100 | Prot_kinase_dom |
|  |  | HORVU2Hr1G006120 |  |
|  |  | HORVU2Hr1G006130 | Glycoside_hydrolase_SF |
|  |  | HORVU2Hr1G006160 |  |
|  |  | HORVU2Hr1G006170 | Glycoside_hydrolase_SF |
|  |  | HORVU2Hr1G006280 |  |
|  |  | HORVU2Hr1G006320 | His_Pase_superF_clade-1 |
|  |  | HORVU2Hr1G006330 | LexA/Signal_pep-like_sf |
|  |  | HORVU2Hr1G006340 | LRR_dom_sf |
|  |  | HORVU2Hr1G006350 | Myb_dom_plants |
|  |  | HORVU2Hr1G006360 | PGG_dom |
|  |  | HORVU2Hr1G006370 | AP2/ERF_dom |
|  |  | HORVU2Hr1G006460 | Transferase |
|  |  | HORVU2Hr1G006470 | LRR_dom_sf |
|  |  | HORVU2Hr1G006480 | NB-ARC |
|  |  | HORVU2Hr1G006490 | LRR_dom_sf |
|  |  | HORVU2Hr1G006500 | NB-ARC |
|  |  | HORVU2Hr1G006510 | SWEET_sugar_transpr |
|  |  | HORVU2Hr1G006520 | SWEET_sugar_transpr |
|  |  | HORVU2Hr1G006530 | Jacalin-like_lectin_dom |
|  |  | HORVU2Hr1G006540 | Transferase |
|  |  | HORVU2Hr1G006560 |  |
|  |  | HORVU2Hr1G006590 | LRR_dom_sf |
|  |  | HORVU2Hr1G006600 | WRKY_dom |
|  |  | HORVU2Hr1G006620 | LRR_dom_sf |
|  |  | HORVU2Hr1G006690 | FH2_Formin |
|  |  | HORVU2Hr1G006700 | Chromo/chromo_shadow_dom |
|  |  | HORVU2Hr1G006720 |  |
|  |  | HORVU2Hr1G006760 |  |
|  |  | HORVU2Hr1G006770 | ELMO_dom |
|  |  | HORVU2Hr1G006810 | DUF677 |
|  |  | HORVU2Hr1G006820 | POT_fam |
|  |  | HORVU2Hr1G006830 | Cyt_P450 |
|  |  | HORVU2Hr1G006850 | Cyt_P450 |
|  |  | HORVU2Hr1G006900 | SAM-dependent_MTases |
|  |  | HORVU2Hr1G006910 | Cyt_P450 |
|  |  | HORVU2Hr1G006930 | Pex16 |
|  |  | HORVU2Hr1G006970 |  |
|  |  | HORVU2Hr1G006980 |  |
|  |  | HORVU2Hr1G006990 | Fatty_acid_desaturase-2 |
|  |  | HORVU2Hr1G007000 | Arsenite_MeTrfase-like |
|  |  | HORVU2Hr1G007030 | Amino_oxidase |
|  |  | HORVU2Hr1G007070 | Amino_oxidase |
|  |  | HORVU2Hr1G007080 | NTF2-like_dom_sf |
|  |  | HORVU2Hr1G007130 | MFS_sugar_transport-like |
|  |  | HORVU2Hr1G007170 | NB-ARC |
|  |  | HORVU2Hr1G007230 | MFS_sugar_transport-like |
|  |  | HORVU2Hr1G007250 | Prot_kinase_dom |
|  |  | HORVU2Hr1G007260 | MFS_sugar_transport-like |
|  |  | HORVU2Hr1G007320 | Kinase-like_dom_sf |
|  |  | HORVU2Hr1G007340 | Prot_kinase_dom |
|  |  | HORVU2Hr1G007350 | FPF1 |
|  |  | HORVU2Hr1G007400 |  |
|  |  | HORVU2Hr1G007420 | WAK_GUB |
|  |  | HORVU2Hr1G007490 | FPF1 |
|  |  | HORVU2Hr1G007500 |  |
|  |  | HORVU2Hr1G007510 |  |
|  |  | HORVU2Hr1G007520 |  |
|  |  | HORVU2Hr1G007530 |  |
|  |  | HORVU2Hr1G007540 |  |
|  |  | HORVU2Hr1G007570 | Methyltransf_11 |
|  |  | HORVU2Hr1G007580 |  |
|  |  | HORVU2Hr1G007590 |  |
|  |  | HORVU2Hr1G007600 | Terpene_synthase_metal-bd |
|  |  | HORVU2Hr1G007610 | NB-ARC |
|  |  | HORVU2Hr1G007640 |  |
|  |  | HORVU2Hr1G007650 | Scorpion_toxin-like |
|  |  | HORVU2Hr1G007660 | NiRdtase/SiRdtase_haem-b_fer |
|  |  | HORVU2Hr1G007670 |  |
|  |  | HORVU2Hr1G007680 | Scorpion_toxin-like |
|  |  | HORVU2Hr1G007710 | Terpene_synth_N |
|  |  | HORVU2Hr1G007720 | O_MeTrfase_dom |
|  |  | HORVU2Hr1G007730 | POT_fam |
|  |  | HORVU2Hr1G007770 |  |
|  |  | HORVU2Hr1G007780 |  |
|  |  | HORVU2Hr1G007800 | NB-ARC |
|  |  | HORVU2Hr1G007810 |  |
|  |  | HORVU2Hr1G007830 |  |
|  |  | HORVU2Hr1G007840 | Chaprnin_Cpn60 |
|  |  | HORVU2Hr1G007950 |  |
|  |  | HORVU2Hr1G007980 | PPIase_FKBP_dom |
|  |  | HORVU2Hr1G007990 | Sulfotransferase_dom |
|  |  | HORVU2Hr1G008000 | Dirigent |
|  |  | HORVU2Hr1G008010 | RMI2 |
|  |  | HORVU2Hr1G008030 |  |
|  |  | HORVU2Hr1G008090 | AA_transpt_TM |
|  |  | HORVU2Hr1G008120 | Dirigent |
|  |  | HORVU2Hr1G008130 | S_locus_glycoprot_dom |
|  |  | HORVU2Hr1G008140 | Prot_kinase_dom |
|  |  | HORVU2Hr1G008220 | S_locus_glycoprot_dom |
|  |  | HORVU2Hr1G008230 | PLAC_motif_containing |
|  |  | HORVU2Hr1G008240 | Prot_kinase_dom |
|  |  | HORVU2Hr1G008250 | Pumilio_RNA-bd_rpt |
|  |  | HORVU2Hr1G008270 | BBE |
|  |  | HORVU2Hr1G008290 |  |
|  |  | HORVU2Hr1G008300 | Oxid_FAD_bind_N |
|  |  | HORVU2Hr1G008310 | Oxid_FAD_bind_N |
|  |  | HORVU2Hr1G008330 | Oxid_FAD_bind_N |
|  |  | HORVU2Hr1G008370 | Kinase-like_dom_sf |
|  |  | HORVU2Hr1G008380 | PPIase_FKBP_dom |
|  |  | HORVU2Hr1G008390 | Bulb-type_lectin_dom |
|  |  | HORVU2Hr1G008410 |  |
|  |  | HORVU2Hr1G008440 | CobW/HypB/UreG_dom |
|  |  | HORVU2Hr1G008570 |  |
|  |  | HORVU2Hr1G008640 | Bulb-type_lectin_dom |
|  |  | HORVU2Hr1G008720 |  |
|  |  | HORVU2Hr1G008740 | Dirigent |
|  |  | HORVU2Hr1G008770 |  |
|  |  | HORVU2Hr1G008890 |  |
|  |  | HORVU2Hr1G008960 | Sulfotransferase_dom |
|  |  | HORVU2Hr1G008980 | UDP_glucos_trans |
|  |  | HORVU2Hr1G008990 | Chalcone/stilbene_synthase_N |
|  |  | HORVU2Hr1G009070 | Znf_PMZ |
|  |  | HORVU2Hr1G009110 | NAM-associated_C |
|  |  | HORVU2Hr1G009150 | Peptidase_C1A_C |
|  |  | HORVU2Hr1G009160 | LRR_dom_sf |
|  |  | HORVU2Hr1G009260 | PP2C |
|  |  | HORVU2Hr1G009280 |  |
|  |  | HORVU2Hr1G009300 | RNaseH_domain |
|  |  | HORVU2Hr1G009450 |  |
|  |  | HORVU2Hr1G009460 |  |
|  |  | HORVU2Hr1G009480 |  |
|  |  | HORVU2Hr1G009490 | PP2C |
|  |  | HORVU2Hr1G009510 | Prot_inhib_I29 |
|  |  | HORVU2Hr1G009520 |  |
|  |  | HORVU2Hr1G009540 | Peptidase_C1A_C |
|  |  | HORVU2Hr1G009560 | FBD |
|  |  | HORVU2Hr1G009570 | FBD |
|  |  | HORVU2Hr1G009580 | ABC_transporter-like |
|  |  | HORVU2Hr1G009610 | Pept_cys_AS |
|  |  | HORVU2Hr1G009640 | Pept_cys_AS |
|  |  | HORVU2Hr1G009680 | ABC_transporter-like |
|  |  | HORVU2Hr1G009710 | Peptidase_C65_otubain |
|  |  | HORVU2Hr1G009730 |  |
|  |  | HORVU2Hr1G009760 |  |
|  |  | HORVU2Hr1G009780 | Gdt1 |
|  |  | HORVU2Hr1G009790 | Legume_lectin_dom |
|  |  | HORVU2Hr1G009800 | Prot_kinase_dom |
|  |  | HORVU2Hr1G009830 | ABC1_TM_dom |
|  |  | HORVU2Hr1G009840 | Vps54 |
|  |  | HORVU2Hr1G009850 |  |
|  |  | HORVU2Hr1G009890 | Sec1-like |
|  |  | HORVU2Hr1G009900 | Flavin_mOase-like |
|  |  | HORVU2Hr1G009940 | Haem_peroxidase |
|  |  | HORVU2Hr1G009950 | EGF-type_Asp/Asn_hydroxyl_site |
|  |  | HORVU2Hr1G009970 | Aspartic_peptidase_A1 |
|  |  | HORVU2Hr1G009980 | Aspartic_peptidase_A1 |
|  |  | HORVU2Hr1G010000 | Arg_decrbxlase |
|  |  | HORVU2Hr1G010100 | Glyas_Fos-R_dOase_dom |
|  |  | HORVU2Hr1G010130 | G3P_DH_FAD-dep |
|  |  | HORVU2Hr1G010140 | UDP_glucos_trans |
|  |  | HORVU2Hr1G010180 |  |
|  |  | HORVU2Hr1G010250 | GroES-like_sf |
|  |  | HORVU2Hr1G010260 | CRAL-TRIO_dom |
|  |  | HORVU2Hr1G010270 | GroES-like_sf |
|  |  | HORVU2Hr1G010280 |  |
|  |  | HORVU2Hr1G010290 |  |
|  |  | HORVU2Hr1G010300 |  |
|  |  | HORVU2Hr1G010320 | Dirigent |
|  |  | HORVU2Hr1G010350 |  |
|  |  | HORVU2Hr1G010380 | Pept_M24 |
|  |  | HORVU2Hr1G010400 | DUF295 |
|  |  | HORVU2Hr1G010430 | Dirigent |
|  |  | HORVU2Hr1G010440 | Biotin_lipoyl |
|  |  | HORVU2Hr1G010450 | SANT/Myb |
|  |  | HORVU2Hr1G010480 | Ribosomal_L30_bac-type |
|  |  | HORVU2Hr1G010490 | Ser-Thr/Tyr_kinase_cat_dom |
|  |  | HORVU2Hr1G010500 | Leu-rich_rpt |
|  |  | HORVU2Hr1G010520 | Aspartic_peptidase_A1 |
|  |  | HORVU2Hr1G010540 | Thioredoxin-like_sf |
|  |  | HORVU2Hr1G010560 | Thioredoxin-like_sf |
|  |  | HORVU2Hr1G010590 | Kinase-like_dom_sf |
|  |  | HORVU2Hr1G010610 | Prot_kinase_dom |
|  |  | HORVU2Hr1G010620 | Prot_kinase_dom |
|  |  | HORVU2Hr1G010630 | RuBisCO_sc_dom |
|  |  | HORVU2Hr1G010660 |  |
|  |  | HORVU2Hr1G010670 | RuBisCO_sc_dom |
|  |  | HORVU2Hr1G010690 | RuBisCO_sc_dom |
|  |  | HORVU2Hr1G010720 |  |
|  |  | HORVU2Hr1G010750 | ICR |
|  |  | HORVU2Hr1G010780 |  |
|  |  | HORVU2Hr1G010800 |  |
|  |  | HORVU2Hr1G010830 | ADH_Zn_CS |
|  |  | HORVU2Hr1G010840 | ADH_Zn_CS |
|  |  | HORVU2Hr1G010860 |  |
|  |  | HORVU2Hr1G010870 | Ribosomal_S6e |
|  |  | HORVU2Hr1G010880 |  |
|  |  | HORVU2Hr1G010890 | Ribosomal_S6e |
|  |  | HORVU2Hr1G010920 | NB-ARC |
|  |  | HORVU2Hr1G010930 | Oxoglu/Fe-dep_dioxygenase |
|  |  | HORVU2Hr1G010950 | Oxoglu/Fe-dep_dioxygenase |
|  |  | HORVU2Hr1G010960 | Oxoglu/Fe-dep_dioxygenase |
|  |  | HORVU2Hr1G010970 | Znf_RING |
|  |  | HORVU2Hr1G010980 | mRNA_cap_enzyme |
|  |  | HORVU2Hr1G010990 | MIP |
|  |  | HORVU2Hr1G011010 | Methyl_CpG_DNA-bd |
|  |  | HORVU2Hr1G011030 | Glutaredoxin |
|  |  | HORVU2Hr1G011040 | HECT_dom |
|  |  | HORVU2Hr1G011060 | HMA_dom |
|  |  | HORVU2Hr1G011080 |  |
|  |  | HORVU2Hr1G011100 |  |
|  |  | HORVU2Hr1G011110 | F-box_dom |
|  |  | HORVU2Hr1G011120 | LRR_dom_sf |
|  |  | HORVU2Hr1G011140 |  |
|  |  | HORVU2Hr1G011160 | Transferase |
|  |  | HORVU2Hr1G011170 | Cyt_P450 |
|  |  | HORVU2Hr1G011180 | F-box_dom |
|  |  | HORVU2Hr1G011200 | Ribosome-assoc_YjgA |
|  |  | HORVU2Hr1G011250 | SANT/Myb |
|  |  | HORVU2Hr1G011270 | SANT/Myb |
|  |  | HORVU2Hr1G011280 |  |
|  |  | HORVU2Hr1G011300 | PB1_dom |
|  |  | HORVU2Hr1G011340 | Cpn60/TCP-1 |
|  |  | HORVU2Hr1G011350 | Pentatricopeptide_repeat |
|  |  | HORVU2Hr1G011360 | NAC-dom |
|  |  | HORVU2Hr1G011370 | NAC-dom |
|  |  | HORVU2Hr1G011390 | NAC-dom |
|  |  | HORVU2Hr1G011400 | Pentatricopeptide_repeat |
|  |  | HORVU2Hr1G011420 |  |
|  |  | HORVU2Hr1G011430 |  |
|  |  | HORVU2Hr1G011450 | Thioredoxin-like_sf |
|  |  | HORVU2Hr1G011460 |  |
|  |  | HORVU2Hr1G011470 |  |
|  |  | HORVU2Hr1G011480 |  |
|  |  | HORVU2Hr1G011490 |  |
|  |  | HORVU2Hr1G011530 |  |
|  |  | HORVU2Hr1G011550 | GH10 |
|  |  | HORVU2Hr1G011560 | WD40_repeat |
|  |  | HORVU2Hr1G011570 |  |
|  |  | HORVU2Hr1G011580 | Cleavage/polyA-sp_fac_asu_C |
|  |  | HORVU2Hr1G011610 | Sec1-like |
|  |  | HORVU2Hr1G011640 |  |
|  |  | HORVU2Hr1G011650 |  |
|  |  | HORVU2Hr1G011660 | Oxoglu/Fe-dep_dioxygenase |
|  |  | HORVU2Hr1G011670 | Oxoglu/Fe-dep_dioxygenase |
|  |  | HORVU2Hr1G011680 | Cyt_P450 |
|  |  | HORVU2Hr1G011690 | AAA+_ATPase |
|  |  | HORVU2Hr1G011700 | RRM_dom |
|  |  | HORVU2Hr1G011720 | Cyt_P450 |
|  |  | HORVU2Hr1G011790 | Cyt_P450 |
|  |  | HORVU2Hr1G011810 |  |
|  |  | HORVU2Hr1G011820 | Cyt_P450 |
|  |  | HORVU2Hr1G011840 | DUF4283 |
|  |  | HORVU2Hr1G011870 | AA-permease/SLC12A_dom |
|  |  | HORVU2Hr1G011910 | Neprosin |
|  |  | HORVU2Hr1G011930 | Cys/Met-Metab_PyrdxlP-dep_enz |
|  |  | HORVU2Hr1G011960 | HSP_DnaJ_Cys-rich_dom_sf |
|  |  | HORVU2Hr1G011970 | Cys/Met-Metab_PyrdxlP-dep_enz |
|  |  | HORVU2Hr1G011980 | Cys/Met-Metab_PyrdxlP-dep_enz |
|  |  | HORVU2Hr1G011990 | Cys/Met-Metab_PyrdxlP-dep_enz |
|  |  | HORVU2Hr1G012010 | Jacalin-like_lectin_dom |
|  |  | HORVU2Hr1G012040 | HSP_DnaJ_Cys-rich_dom_sf |
|  |  | HORVU2Hr1G012060 | Pept_M24 |
|  |  | HORVU2Hr1G012080 | Ribosomal_S14 |
|  |  | HORVU2Hr1G012100 | Clathrin_L-chain |
|  |  | HORVU2Hr1G012110 | Peptidase_C1A_C |
|  |  | HORVU2Hr1G012130 | Wax_synthase |
|  |  | HORVU2Hr1G012140 | Leu-rich_rpt |
|  |  | HORVU2Hr1G012160 |  |
|  |  | HORVU2Hr1G012180 |  |
|  |  | HORVU2Hr1G012200 | C2_dom |
|  |  | HORVU2Hr1G012210 | Pentatricopeptide_repeat |
|  |  | HORVU2Hr1G012220 |  |
|  |  | HORVU2Hr1G012230 | Palmitoyltrfase_DHHC |
|  |  | HORVU2Hr1G012250 | O-acyltransferase_WSD1_N |
|  |  | HORVU2Hr1G012260 | O-acyltransferase_WSD1_C |
|  |  | HORVU2Hr1G012270 | O-acyltransferase_WSD1_C |
|  |  | HORVU2Hr1G012280 |  |
|  |  | HORVU2Hr1G012300 | 5_3_exoribonuclease |
|  |  | HORVU2Hr1G012310 | F-box_dom |
|  |  | HORVU2Hr1G012320 |  |
|  |  | HORVU2Hr1G012340 | UDP_glucos_trans |
|  |  | HORVU2Hr1G012360 | P-loop_NTPase |
|  |  | HORVU2Hr1G012380 |  |
|  |  | HORVU2Hr1G012400 | LRR_dom_sf |
|  |  | HORVU2Hr1G012430 |  |
|  |  | HORVU2Hr1G012440 | Biotin_lipoyl |
|  |  | HORVU2Hr1G012460 |  |
|  |  | HORVU2Hr1G012480 |  |
|  |  | HORVU2Hr1G012500 |  |
|  |  | HORVU2Hr1G012510 |  |
|  |  | HORVU2Hr1G012520 | Peptidase_C65_otubain |
|  |  | HORVU2Hr1G012540 | Peptidase_C65_otubain |
|  |  | HORVU2Hr1G012550 |  |
|  |  | HORVU2Hr1G012580 | LRR_dom_sf |
|  |  | HORVU2Hr1G012600 | Fatty_acid_desaturase-2 |
|  |  | HORVU2Hr1G012630 | Glyco_trans_35 |
|  |  | HORVU2Hr1G012650 | TPP_enzyme-bd_C |
|  |  | HORVU2Hr1G012660 | TPP_enzyme-bd_C |
|  |  | HORVU2Hr1G012670 | At4g02000-like |
|  |  | HORVU2Hr1G012680 |  |
|  |  | HORVU2Hr1G012690 | Band_7 |
|  |  | HORVU2Hr1G012700 | Mycolic_cyclopropane_synthase |
|  |  | HORVU2Hr1G012710 | Cu_amine_oxidase |
|  |  | HORVU2Hr1G012770 | 3'-5'_exonuclease_dom |
|  |  | HORVU2Hr1G012780 | Fatty_acid_desaturase-2 |
|  |  | HORVU2Hr1G012790 | Transketolase_N |
|  |  | HORVU2Hr1G012810 | Cyt_P450 |
|  |  | HORVU2Hr1G012830 | Lactate/malate_DH_N |
|  |  | HORVU2Hr1G012840 | HMGI/Y_DNA-bd_CS |
|  |  | HORVU2Hr1G012850 | Znf_CCHC |
|  |  | HORVU2Hr1G012860 | TF_GTP-bd_dom |
|  |  | HORVU2Hr1G012920 |  |
|  |  | HORVU2Hr1G012930 | Sirtuin |
|  |  | HORVU2Hr1G012940 | Transferase |
|  |  | HORVU2Hr1G012970 | 6-blade_b-propeller_TolB-like |
|  |  | HORVU2Hr1G012980 | Cyt_P450 |
|  |  | HORVU2Hr1G013020 | PC-Esterase |
|  |  | HORVU2Hr1G013060 | Znf_Dof |
|  |  | HORVU2Hr1G013080 | Prot_kinase_dom |
|  |  | HORVU2Hr1G013090 | SANT/Myb |
|  |  | HORVU2Hr1G013110 | MIP |
|  |  | HORVU2Hr1G013130 | Prot_kinase_dom |
|  |  | HORVU2Hr1G013150 |  |
|  |  | HORVU2Hr1G013170 | DH_E1 |
|  |  | HORVU2Hr1G013180 |  |
|  |  | HORVU2Hr1G013200 | LITAF |
|  |  | HORVU2Hr1G013210 | Pec_lyase |
|  |  | HORVU2Hr1G013230 | PDRG1 |
|  |  | HORVU2Hr1G013250 |  |
|  |  | HORVU2Hr1G013260 | Glutaredoxin-like |
|  |  | HORVU2Hr1G013270 | DUF3444 |
|  |  | HORVU2Hr1G013300 | Rpf2 |
|  |  | HORVU2Hr1G013330 |  |
|  |  | HORVU2Hr1G013340 | LolPI |
|  |  | HORVU2Hr1G013350 |  |
|  |  | HORVU2Hr1G013360 | LolPI |
|  |  | HORVU2Hr1G013370 | NAB |
|  |  | HORVU2Hr1G013380 | LolPI |
|  |  | HORVU2Hr1G013390 | UDP_glucos_trans |
|  |  | HORVU2Hr1G013400 | Sig_transdc_resp-reg_receiver |
|  |  | HORVU2Hr1G013420 | Znf_RING |
|  |  | HORVU2Hr1G013440 | Prot_kinase_dom |
|  |  | HORVU2Hr1G013450 | bHLH_dom |
|  |  | HORVU2Hr1G013460 | 6-blade_b-propeller_TolB-like |
|  |  | HORVU2Hr1G013470 | AV_PCPalpha |
|  |  | HORVU2Hr1G013480 | DUF155 |
|  |  | HORVU2Hr1G013500 | Preph_deHydtase |
|  |  | HORVU2Hr1G013520 | Glyco_trans_43 |
|  |  | HORVU2Hr1G013550 |  |
|  |  | HORVU2Hr1G013560 | RuvA_2-like |
|  |  | HORVU2Hr1G013570 |  |
|  |  | HORVU2Hr1G013590 | Glyco_trans_43 |
|  |  | HORVU2Hr1G013620 |  |
|  |  | HORVU2Hr1G013630 | Glyco_trans_43 |
|  |  | HORVU2Hr1G013680 | TF_GTP-bd_dom |
|  |  | HORVU2Hr1G013700 |  |
|  |  | HORVU2Hr1G013710 | Leu-rich_rpt |
|  |  | HORVU2Hr1G013730 | C2_dom |
|  |  | HORVU2Hr1G013740 | Peroxidase_pln |
|  |  | HORVU2Hr1G013760 | Mlo |
|  |  | HORVU2Hr1G013790 | ARM-like |
|  |  | HORVU2Hr1G013810 |  |
|  |  | HORVU2Hr1G013820 | DUF4228_plant |
|  |  | HORVU2Hr1G013830 | FHY3/FAR1 |
|  |  | HORVU2Hr1G013840 |  |
|  |  | HORVU2Hr1G013850 |  |
|  |  | HORVU2Hr1G013870 | FA_desaturase_dom |
|  |  | HORVU2Hr1G013900 | WD40_repeat |
|  |  | HORVU2Hr1G013920 | Jacalin-like_lectin_dom |
|  |  | HORVU2Hr1G013930 | Oxid_FAD_bind_N |
|  |  | HORVU2Hr1G013940 | Jacalin-like_lectin_dom |
|  |  | HORVU2Hr1G013960 | RNR_lg_C |
|  |  | HORVU2Hr1G013970 |  |
|  |  | HORVU2Hr1G013990 | RNR_lg_C |
|  |  | HORVU2Hr1G014000 | PSMC4 |
|  |  | HORVU2Hr1G014010 | Znf_TTF |
|  |  | HORVU2Hr1G014020 | Jacalin-like_lectin_dom |
|  |  | HORVU2Hr1G014030 | LRR_dom_sf |
|  |  | HORVU2Hr1G014050 | PSMC4 |
|  |  | HORVU2Hr1G014060 | PSMC4 |
|  |  | HORVU2Hr1G014080 | PSMC4 |
|  |  | HORVU2Hr1G014120 |  |
|  |  | HORVU2Hr1G014130 | A/AMP_deaminase_dom |
|  |  | HORVU2Hr1G014140 | PC-Esterase |
|  |  | HORVU2Hr1G014150 | PP2C_BS |
|  |  | HORVU2Hr1G014230 | ACP-like_sf |
|  |  | HORVU2Hr1G014240 | NmrA-like |
|  |  | HORVU2Hr1G014260 | ACP-like_sf |
|  |  | HORVU2Hr1G014270 | ACP-like_sf |
|  |  | HORVU2Hr1G014300 | F-box_dom |
|  |  | HORVU2Hr1G014360 | Thiamin_PyroP_enz_TPP |
|  |  | HORVU2Hr1G014380 |  |
|  |  | HORVU2Hr1G014390 | Thiamin_PyroP_enz |
|  |  | HORVU2Hr1G014410 |  |
|  |  | HORVU2Hr1G014440 | ThiF_NAD_FAD-bd |
|  |  | HORVU2Hr1G014450 | Kinase-like_dom_sf |
|  |  | HORVU2Hr1G014480 | Bulb-type_lectin_dom |
|  |  | HORVU2Hr1G014490 |  |
|  |  | HORVU2Hr1G014500 | CRISP-related |
|  |  | HORVU2Hr1G014530 | GH16 |
|  |  | HORVU2Hr1G014550 |  |
|  |  | HORVU2Hr1G014560 |  |
|  |  | HORVU2Hr1G014580 | S1_domain |
|  |  | HORVU2Hr1G014590 | HSF_fam |
|  |  | HORVU2Hr1G014600 |  |
|  |  | HORVU2Hr1G014610 |  |
|  |  | HORVU2Hr1G014660 | SNF2_N |
|  |  | HORVU2Hr1G014670 |  |
|  |  | HORVU2Hr1G014740 |  |
|  |  | HORVU2Hr1G014760 |  |
|  |  | HORVU2Hr1G014770 | ATPase_AAA_core |
|  |  | HORVU2Hr1G014840 | Prot_kinase_dom |
|  |  | HORVU2Hr1G014860 | SCAR/WAVE_fam |
|  |  | HORVU2Hr1G014870 | Znf_C2H2_type |
|  |  | HORVU2Hr1G014890 | Prot_kinase_dom |
|  |  | HORVU2Hr1G014900 | Prot_kinase_dom |
|  |  | HORVU2Hr1G014920 |  |
|  |  | HORVU2Hr1G014930 | Prot_kinase_dom |
|  |  | HORVU2Hr1G014950 |  |
|  |  | HORVU2Hr1G014980 | Nucl-diP-sugar_transferase |
|  |  | HORVU2Hr1G015010 | Legume_lectin_dom |
|  |  | HORVU2Hr1G015020 |  |
|  |  | HORVU2Hr1G015080 | NA-bd_OB-fold |
|  |  | HORVU2Hr1G015100 | Znf_PMZ |
|  |  | HORVU2Hr1G015110 | Gprotein_alpha_su |
|  |  | HORVU2Hr1G015140 | SDR_fam |
|  |  | HORVU2Hr1G015150 | SDR_fam |
|  |  | HORVU2Hr1G015160 | SDR_fam |
|  |  | HORVU2Hr1G015170 |  |
|  |  | HORVU2Hr1G015180 | SDR_fam |
|  |  | HORVU2Hr1G015190 | SDR_fam |
|  |  | HORVU2Hr1G015200 |  |
|  |  | HORVU2Hr1G015240 |  |
|  |  | HORVU2Hr1G015260 | Nas_poly-pep-assoc_cplx_dom |
|  |  | HORVU2Hr1G015310 |  |
|  |  | HORVU2Hr1G015320 |  |
|  |  | HORVU2Hr1G015340 | Prot_kinase_dom |
|  |  | HORVU2Hr1G015360 | Wun1-like |
|  |  | HORVU2Hr1G015380 | Prot_kinase_dom |
|  |  | HORVU2Hr1G015410 | Wun1-like |
|  |  | HORVU2Hr1G015430 | D-Ala_lig_Van_CS |
|  |  | HORVU2Hr1G015440 | COBRA_pln |
|  |  | HORVU2Hr1G015470 | PP2C |
|  |  | HORVU2Hr1G015480 | PPM-type_phosphatase_dom |
|  |  | HORVU2Hr1G015510 |  |
|  |  | HORVU2Hr1G015520 | Cyt_c_oxidase_su2_TM_dom |
|  |  | HORVU2Hr1G015530 | Cyt_c_oxidase-like_su3 |
|  |  | HORVU2Hr1G015540 | NADH_Q_OxRdtase_suD |
|  |  | HORVU2Hr1G015560 |  |
|  |  | HORVU2Hr1G015570 | ACT_dom |
|  |  | HORVU2Hr1G015590 | ACT_dom |
|  |  | HORVU2Hr1G015620 | Pectinesterase_cat |
|  |  | HORVU2Hr1G015670 | Znf_PHD |
|  |  | HORVU2Hr1G015690 |  |
|  |  | HORVU2Hr1G015700 | Glyco_trans_8 |
|  |  | HORVU2Hr1G015710 |  |
|  |  | HORVU2Hr1G015720 | Glyco_trans_8 |
|  |  | HORVU2Hr1G015740 | Nucleotide-diphossugar_trans |
|  |  | HORVU2Hr1G015760 | BRCT_dom |
|  |  | HORVU2Hr1G015800 |  |
|  |  | HORVU2Hr1G015830 |  |
|  |  | HORVU2Hr1G015850 | DnaJ_domain |
|  |  | HORVU2Hr1G015870 | P-loop_NTPase |
|  |  | HORVU2Hr1G015880 |  |
|  |  | HORVU2Hr1G015890 |  |
|  |  | HORVU2Hr1G015900 | FHY3/FAR1 |
|  |  | HORVU2Hr1G015920 |  |
|  |  | HORVU2Hr1G015930 | Prot_kinase_dom |
|  |  | HORVU2Hr1G015940 | Endosulphine |
|  |  | HORVU2Hr1G015980 | Nicotian_synth |
|  |  | HORVU2Hr1G015990 | 6-blade_b-propeller_TolB-like |
|  |  | HORVU2Hr1G016000 | Lon_substr-bd |
|  |  | HORVU2Hr1G016030 | Leu-rich_rpt |
|  |  | HORVU2Hr1G016040 | Aldehyde_DH_dom |
|  |  | HORVU2Hr1G016070 |  |
|  |  | HORVU2Hr1G016100 | Polymerase_NTP_transf_dom |
|  |  | HORVU2Hr1G016120 | TF_GRAS |
|  |  | HORVU2Hr1G016150 |  |
|  |  | HORVU2Hr1G016160 |  |
|  |  | HORVU2Hr1G016180 |  |
|  |  | HORVU2Hr1G016200 | SASA |
|  |  | HORVU2Hr1G016220 |  |
|  |  | HORVU2Hr1G016230 |  |
|  |  | HORVU2Hr1G016250 | DUF1618 |
|  |  | HORVU2Hr1G016290 |  |
|  |  | HORVU2Hr1G016300 | Armadillo |
|  |  | HORVU2Hr1G016310 |  |
|  |  | HORVU2Hr1G016330 | RAD52-like |
|  |  | HORVU2Hr1G016380 | SANT/Myb |
|  |  | HORVU2Hr1G016410 | RAD52-like |
|  |  | HORVU2Hr1G016430 | SPEAR |
|  |  | HORVU2Hr1G016440 | Pentatricopeptide_repeat |
|  |  | HORVU2Hr1G016470 |  |
|  |  | HORVU2Hr1G016510 | PPIase_PpiC |
|  |  | HORVU2Hr1G016540 | DUF295 |
|  |  | HORVU2Hr1G016560 | SGNH_hydro_sf |
|  |  | HORVU2Hr1G016580 | bZIP |
|  |  | HORVU2Hr1G016630 | Frigida |
|  |  | HORVU2Hr1G016650 | GTP_binding_domain |
|  |  | HORVU2Hr1G016660 |  |
|  |  | HORVU2Hr1G016690 |  |
|  |  | HORVU2Hr1G016720 | Prot_kinase_dom |
|  |  | HORVU2Hr1G016730 | ASC_dom |
|  |  | HORVU2Hr1G016740 | FSH_dom |
|  |  | HORVU2Hr1G016750 | Prot_kinase_dom |
|  |  | HORVU2Hr1G016760 | Alpha-L-AF_C |
|  |  | HORVU2Hr1G016780 |  |
|  |  | HORVU2Hr1G016800 | RINT1_Tip20 |
|  |  | HORVU2Hr1G016840 | DAHP1/KDSA |
|  |  | HORVU2Hr1G016860 |  |
|  |  | HORVU2Hr1G016910 |  |
|  |  | HORVU2Hr1G016940 |  |
|  |  | HORVU2Hr1G016990 |  |
|  |  | HORVU2Hr1G017020 | bZIP |
|  |  | HORVU2Hr1G017040 | Peptidase_S8/S53_dom |
|  |  | HORVU2Hr1G017080 | AB_hydrolase_1 |
|  |  | HORVU2Hr1G017090 | Prot_kinase_dom |
|  |  | HORVU2Hr1G017100 | Peptidase_S8/S53_dom |
|  |  | HORVU2Hr1G017120 | SDR_fam |
|  |  | HORVU2Hr1G017170 | ImidazoleglycerolP_deHydtase |
|  |  | HORVU2Hr1G017240 | SANT/Myb |
|  |  | HORVU2Hr1G017270 | Homeobox_dom |
|  |  | HORVU2Hr1G017290 | Znf_Dof |
|  |  | HORVU2Hr1G017310 | Thioredoxin_domain |
|  |  | HORVU2Hr1G017330 | Ribosomal_S16 |
|  |  | HORVU2Hr1G017340 | Ribosomal_S19 |
|  |  | HORVU2Hr1G017350 | NAC-dom |
|  |  | HORVU2Hr1G017360 | NAC-dom |
|  |  | HORVU2Hr1G017370 | NAC-dom |
|  |  | HORVU2Hr1G017380 | NAC-dom |
|  |  | HORVU2Hr1G017400 | NAC-dom |
|  |  | HORVU2Hr1G017410 |  |
|  |  | HORVU2Hr1G017420 | Ricin_B-like_lectins |
|  |  | HORVU2Hr1G017430 |  |
|  |  | HORVU2Hr1G017440 | Ricin_B-like_lectins |
|  |  | HORVU2Hr1G017450 |  |
|  |  | HORVU2Hr1G017460 | NAC-dom |
|  |  | HORVU2Hr1G017470 |  |
|  |  | HORVU2Hr1G017490 | RRM_dom |
|  |  | HORVU2Hr1G017500 | NAD(P)-bd_dom_sf |
|  |  | HORVU2Hr1G017530 |  |
|  |  | HORVU2Hr1G017580 |  |
|  |  | HORVU2Hr1G017630 | Prot_kinase_dom |
|  |  | HORVU2Hr1G017640 | Transl_init_fac_IF2/IF5 |
|  |  | HORVU2Hr1G017650 | Prot_kinase_dom |
|  |  | HORVU2Hr1G017680 | SNF2_N |
|  |  | HORVU2Hr1G017710 |  |
|  |  | HORVU2Hr1G017720 | WRKY_dom |
|  |  | HORVU2Hr1G017740 | WRKY_dom |
|  |  | HORVU2Hr1G017760 | B3_DNA-bd |
|  |  | HORVU2Hr1G017770 | PA domain |
|  |  | HORVU2Hr1G017780 |  |
|  |  | HORVU2Hr1G017790 | Trm1 |
|  |  | HORVU2Hr1G017820 | basic leu zipper domain |
|  |  |  |  |
